# Structural predictions of protein–DNA binding: MELD-DNA

**DOI:** 10.1093/nar/gkad013

**Published:** 2023-02-02

**Authors:** Reza Esmaeeli, Antonio Bauzá, Alberto Perez

**Affiliations:** Department of Chemistry, Quantum theory project, University of Florida, Gainesville, FL 32611, USA; Department of Chemistry, Universitat de les Illes Balears, Palma de Mallorca (Baleares), 07122, Spain; Department of Chemistry, Quantum theory project, University of Florida, Gainesville, FL 32611, USA

## Abstract

Structural, regulatory and enzymatic proteins interact with DNA to maintain a healthy and functional genome. Yet, our structural understanding of how proteins interact with DNA is limited. We present MELD-DNA, a novel computational approach to predict the structures of protein–DNA complexes. The method combines molecular dynamics simulations with general knowledge or experimental information through Bayesian inference. The physical model is sensitive to sequence-dependent properties and conformational changes required for binding, while information accelerates sampling of bound conformations. MELD-DNA can: (i) sample multiple binding modes; (ii) identify the preferred binding mode from the ensembles; and (iii) provide qualitative binding preferences between DNA sequences. We first assess performance on a dataset of 15 protein–DNA complexes and compare it with state-of-the-art methodologies. Furthermore, for three selected complexes, we show sequence dependence effects of binding in MELD predictions. We expect that the results presented herein, together with the freely available software, will impact structural biology (by complementing DNA structural databases) and molecular recognition (by bringing new insights into aspects governing protein–DNA interactions).

## INTRODUCTION

Understanding and predicting how proteins and nucleic acids interact is key to deciphering the mechanisms regulating gene expression, genome repair and storage, with applications in fields such as nanomedicine (1), transition metal chemistry (2) or clinical diagnosis (3), to name a few. The prediction of such molecular recognition problems typically falls under molecular docking, machine learning and molecular dynamics-based studies, which have been broadly successful in understanding how proteins recognize small molecules, peptides and other proteins (4–6). However, the nature of protein–DNA interactions introduces several nuances that have challenged standard approaches. First, whereas proteins come in diverse shapes and sizes, the double-stranded B-DNA structure is common to most DNA sequences, leading for many years to the concept of proteins ‘reading’ DNA. Second, the highly charged interactions from the repeating phosphate backbone lead to a particular protein interacting with high affinity with many different DNA sequences. Despite the high affinity, specificity (7) for different sequences can span several orders of magnitude, leading to a preferential binding for certain sequences. Transcription factor (TF) proteins regulate gene expression by binding DNA sequences between 6 and 12 bp in length, statistically found thousands to millions of times along the genome. Still, most of these sites are never experimentally occupied (8,9). TF binding has received particular attention due to its biological roles; thus, we will focus on those. Understanding TF protein–DNA interactions requires answering three distinct questions: (i) what structure will a particular protein–DNA complex adopt; (ii) what sequence will a particular TF preferentially bind (e.g. where along the genome will the complex form); and (iii) what are the relative binding affinities for different sequences? Pipelines combining elements from structural databases, molecular dynamics, docking and machine learning are becoming more prominent to address some (or all) of the above questions (10,11).

While the initial views on protein–DNA recognition focused on the ability of proteins to ‘read’ the DNA sequence and find the best binding site, current views show that DNA has a much more prominent role in recognition (9,12). Thus, some proteins interact with DNA through sequence-specific interactions, but many interact through shared DNA features (e.g. the phosphate backbone). The difference in binding patterns leads to binding mechanisms lying between two extremes: sequence readout and shape readout (9,13–16). The first is governed by specific interactions, while the second accounts for the ability of a particular DNA sequence to adopt conformations compatible with the structure of the complex. Attempts at combining structural descriptors (e.g. average groove widths, propeller twist or roll) combined with sequence preferences derived from genomic studies (7,17–18) significantly improve predictions of where TF proteins bind along the genome. However, attempts to use detailed structural approaches, such as free energy perturbation or energy decomposition, to distinguish shape and sequence contributions to binding are not always successful, limiting their general application (19–21). Although the reason for failures are unclear, factors such as the amount of DNA deformation in different TFs, the number of binding modes contributing to the binding free energy and force field accuracy are suspected to be involved (21).

We require initial structures of the complex for many studies involving an understanding of binding affinity predictions, binding mechanisms or even DNA-mediated allostery. However, there is a lack of experimental structural data and few computational methods to predict such complexes accurately. For instance, there are 6052 protein–DNA structures in the Nucleic Acid Database, compared with 171 077 protein structures in the Protein Data Bank (PDB) as of October 2022 (22,23). Such small datasets pose challenges to adapting protein structure prediction approaches [e.g. AlphaFold (24)] to the problem of predicting nucleic acids and their complexes. The recent RoseTTAFoldNA (25) machine learning approach compensates for the smaller datasets (they report 1556 protein–nucleic acid complex clusters compared with 26 128 all protein clusters) by incorporating physics-based parameters (e.g. van der Waals terms) originating from Rosetta. Despite the advances this method represents, especially in RNA structure prediction, it requires further development to correct anomalies in predicting structures of homodimers bound to DNA (prevalent in TFs), which leads to an overlap between monomer units and incorrect binding modes.

For many decades, docking has been the most efficient technique for predicting atomic resolution structures of macromolecular binding. (26) Docking is an invaluable tool for the virtual screening of small molecule libraries in the early stages of drug discovery (27) thanks to the balance between computational efficiency and accuracy. For larger assemblies, community efforts such as the Critical Assessment of PRediction of Interactions (CAPRI) (28) have led to significant improvements in the field. However, the accuracy of docking drops rapidly for systems involving conformational changes and in charged systems, where scoring functions are less reliable. Thus, docking methods will often combine with strategies such as normal modes or a later stage using molecular dynamics to overcome limitations in scoring functions (29,30). The most successful efforts come from the High-Ambiguity Driven Docking (HADDOCK) (31,30) group combining docking with ambiguous data and DNA flexibility (via normal modes). They have led to helpful benchmark sets of different difficulties for assessing protein–DNA docking performance (32).

Knowing the structure of the complex opens up the possibility of predicting relative binding affinities for different sequences. Alchemical free energy methods based on molecular dynamics simulations are the gold standard for predicting relative (and absolute) binding affinities for small molecules (33). However, they present limitations when dealing with electrostatic charge variations and flexible complexes where several binding modes can contribute to the binding free energy (34). Despite some successes for protein–DNA systems (35,20), a recent systematic study (21) on protein–DNA complexes points to deficiencies of these approaches arising from either phase space overlap or force field issues. Thus, current methods based on physics and statistical potentials such as Rosetta (36) or machine learning (37–40) far outperform traditional molecular dynamics-based approaches in speed and accuracy.

In this study, we introduce a framework based on alchemical free energy methods that incorporates information via Bayesian inference to predict structure–sequence relationships to increase our understanding of how proteins bind nucleic acids. The approach, MELD (Modeling Employing Limited Data) (41,42), has been previously used for predicting protein structures and their complexes with small molecules, peptides and proteins. Here we extend the framework to DNA (MELD-DNA), with different applications depending on the type of data used. This work exemplifies three of the most common uses: (i) predicting structures of protein–DNA complexes; (ii) identifying sequence sensitivity; and (iii) predicting relative binding affinities. We expect the first application to be the most broadly used and thus present a generalized protocol over 15 different proteins. We make more specific comparisons over a series of 15 sequences for three of those systems. Finally, we exemplify the application of relative binding affinities for six sequences binding a particular TF. The current work represents an extensive simulation study with an aggregated 1 ms of sampling. Our results show that MELD-DNA successfully: (i) samples multiple binding modes; (ii) identifies the experimental binding mode through clustering the ensembles; and (iii) is sensitive to DNA sequences and conformations.

## MATERIALS AND METHODS

### General approach

We use the MELD Bayesian inference approach [*p*(*x|D*) α*p*(*D|x*) · *p*(*x*)] to incorporate ambiguous and noisy data to enhance binding/unbinding events (41,42). The prior distribution [*p*(*x*)] is given by the Boltzmann distribution based on the chosen force field, while the likelihood [*p*(*D|x*)] comes from the agreement of the sampled conformations (*x*) with a subset of the data (*D*, the one with the lowest restraint energy). As MELD samples the energy landscape, different subsets of data are also explored, exploiting regions compatible with some subset of data, and the force field (41,42) gives rise to the posterior distribution [*p*(*x|D*)]. In practical terms, MELD uses a Hamiltonian and Temperature replica exchange molecular dynamics approach in which some replica conditions are compatible with unbound states and some with bound states. As ‘walkers’ sample different conditions in the replica ladder, they go through cycles of binding and unbinding. We identify bound states by clustering the lowest temperature ensembles, where each cluster represents a different binding mode and is compatible with varying subsets of data. We will showcase here three protocols to address three questions: (i) general binding (applied to any protein–DNA system); (ii) specific binding (applied to many DNA sequences binding a particular protein where additional information is known); and (iii) relative binding affinities. The type of data used to guide simulations depends on the questions we ask. Examples are accessible from Zenodo (see Data Availability section). MELD simulations use 30 replicas, the parmBSC1 force field for nucleic acids (43–45), the ff14SB side force field for the protein (46,47) and the GBneck2Nu implicit solvent model (48,49). Throughout all protocols, we include restraints to keep the protein and DNA from unfolding at high temperatures. For proteins, we enforce secondary structure and flat-bottom harmonic restraints on native Cα–Cα contacts; the initial coordinates for simulations and to set up the restraints are based on its bound conformation. For DNA, we implement restraints that maintain hydrogen-bonding patterns at each base pair to prevent DNA melting. All simulations were initialized with the protein far away (at least 30 Å) from the DNA. The initial DNA conformation is generated in its canonical B-form based on the sequence (50).

### Protocol 1: posing general knowledge in terms of ambiguous data drives protein–DNA structure prediction

**Figure 1.**
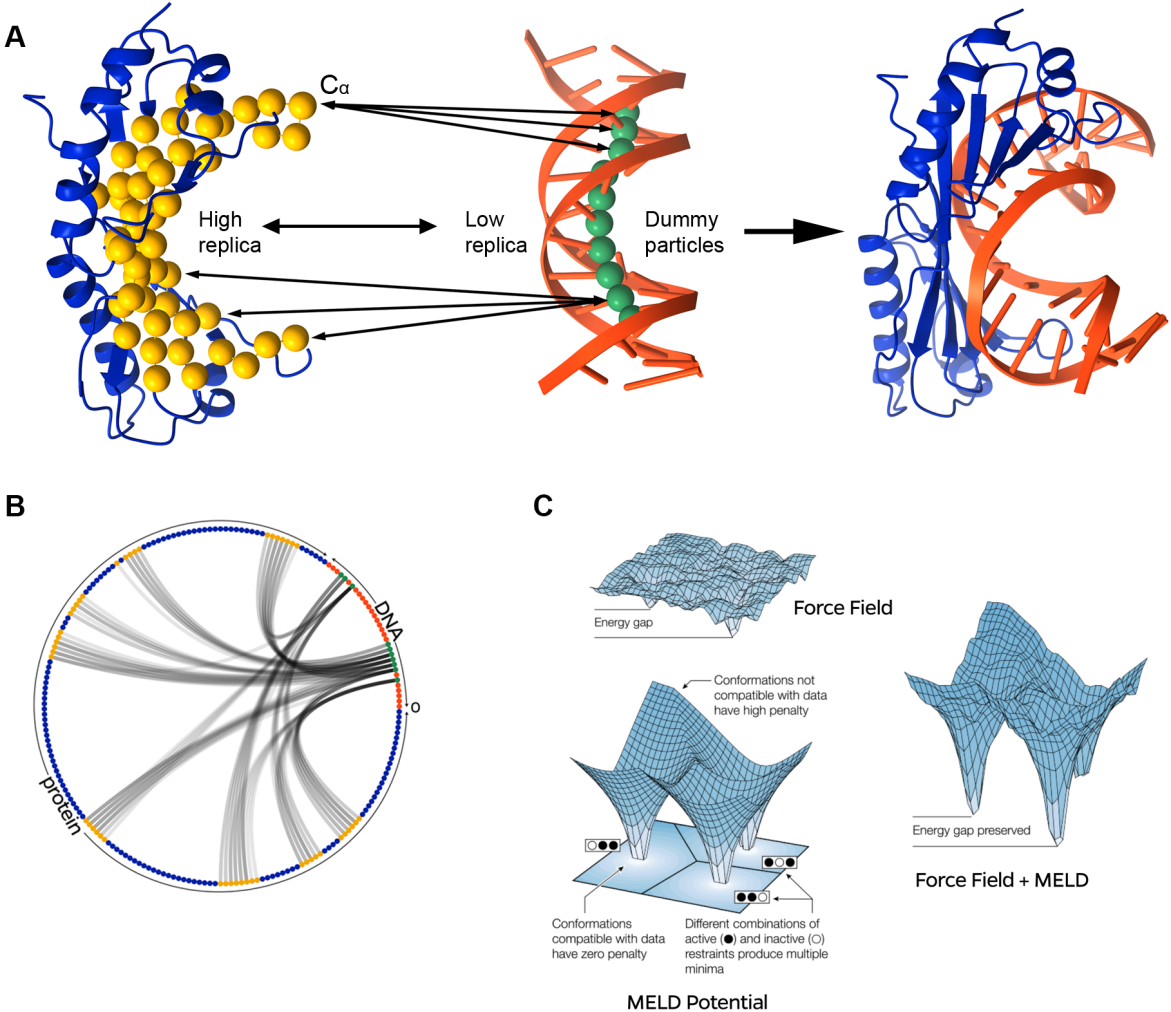
Schematic of the MELD approach. (**A**) Each system starts from an unbound state [minimum of 30 Å distance between the protein (blue) and DNA (orange)]. Information for the simulation is derived from possible interactions between the known interface Cα atoms (yellow) and dummy particles (green) projected on purine N1 atoms. Clustering yields the representative structure on the right. (**B**) Representation of the data in a circular plot where each residue is a dot on the circle, with black lines representing the information used to direct binding. MELD simulations satisfy only a fraction of all the possibilities at any given time of the simulation. (**C**) Schematic of the MELD Bayesian inference approach, adapted from (41).

In this approach, we seek to explore multiple binding modes and rely on statistical mechanics to identify the most native-like one (e.g. the most compatible with our physical model). We presume knowledge of (i) the protein structure, (ii) the DNA sequence to bind and (iii) the DNA-binding domain. We generate a B-DNA structure using Chimera (50) and create a dummy atom at the N1 position of each purine base (see Figure 1). We define the binding data as the possible interactions between Cα atoms in the binding domain and the N1 atoms (Figure 1A). We produce a list of potential contacts, where only some might be satisfied during binding (noisy data) (Figure 1B). We reduce the amount of possible combinatorics by taking into consideration geometric considerations (e.g. residues far away in the binding site are unlikely to interact with the same DNA base simultaneously). Clustering on the MELD ensemble, we identify native-like poses in the ensemble (see Figure 1). The current set-up has two advantages: (i) by using dummy particles at the N1 site, we do not favor the protein approaching through either major or minor groove orientations and (ii) the information added is not exhaustive of all possibilities—and it does not need to be, as the force field will sample the most likely conformations given the available data (Figure 1C).

We chose 15 protein–DNA systems to apply this approach (see Table 1). The systems include complexes with little or no deformation of the DNA from its canonical B-DNA form, others that induce moderate deformation upon binding and complexes where the DNA is far from its canonical B-DNA conformation. The dataset also contains systems that have been solved experimentally with two different sequences, resulting in binding mode variations (e.g. different spacing between binding domains: 1R4R and 1R4O). Finally, we include two types of systems intended to challenge our approach: binding occurs through either flexible (disordered) tails (1ZME) or where large conformational changes are needed for accessing the binding site (1BGB and 2B0D).

**Table 1.** Protein–DNA systems simulated in this study, along with their DNA sequence and PDB IDs

System	DNA sequence	PDB	Reference
Nuclear intron-encoded homing endonuclease I-Ppoi	TGACTCTCTTAAGAGAGTCA	1A74	(51)
Hyperthermophile chromosomal protein Sac7d	GCGATCGC	1AZP	(52)
9-*Cis* retinoic acid receptor	TAGGTCAAAGGTCAG	1BY4	(53)
Human papillomavirus type-18 E2	CAACCGAATTCGGTTG	1JJ4	(54)
Phage 434 Cro	AGTACAAACTTTCTTGTAT	3CRO	(55)
Fungal transcription factor Put3	CGGGAAGCCAACTCCG	1ZME	(56)
Murine Creb Bzip–Cre complex	CTTGGCTGACGTCAGCCAAG	1DH3	(57)
P22 C2 repressor	CATTTAAGATATCTTAAATA	2R1J	(58)
Human Tbp core domain	CTGCTATAAAAGGCTG	1CDW	(59)
Gcn4 leucine zipper	TTCCTATGACTCATCCAGTT	1YSA	(60)
Gcn4 leucine zipper	TGGAGATGACGTCATCTCC	2DGC	(61)
Glucocorticoid eeceptor	TCAGAACATGATGTTCTCA	1R4R	(62)
Glucocorticoid eeceptor	CCAGAACATCGATGTTCTG	1R4O	(62)
EcoRV restriction endonuclease	CGGGATATCCC	1BGB	(63)
EcoRV restriction endonuclease	AAAGAATTCTT	2B0D	(64)

### Protocol 2: system-specific binding protocols tease out sequence effects

**Figure 2.**
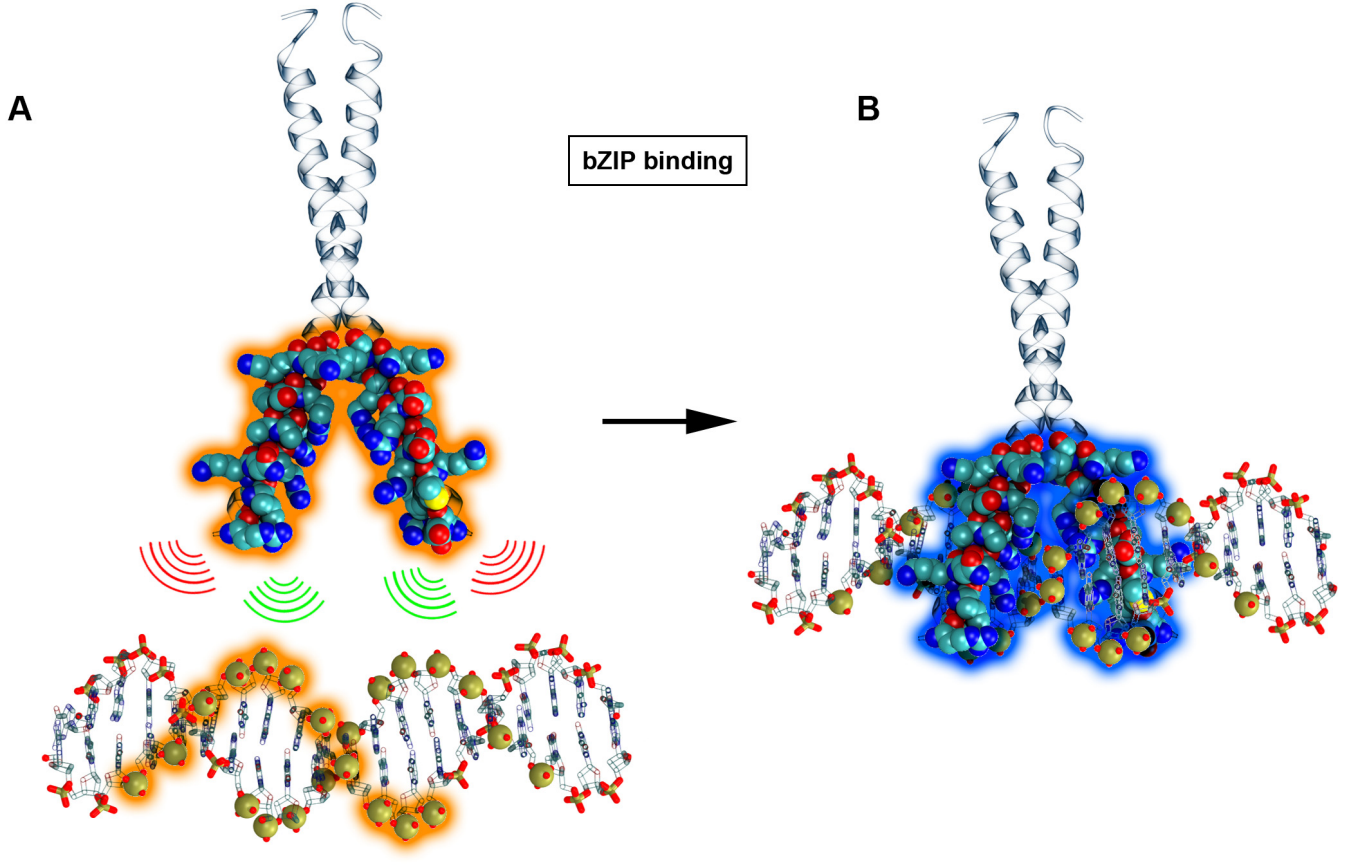
Representation of ambiguity for bZIP binding. (**A**) Phosphate sites along the DNA sequence are combinatorically paired with C_β_ atoms in the binding site of the bZIP protein (highlighted in orange). (**B**) The native interactions from the experimental structure are highlighted with a blue halo.

In this scenario, we have knowledge of the bound state (and binding mode) and use this information to guide to repeating binding sites along a DNA oligomer (see Figure 2). By using oligomers with different sequences (but the same driving information), we are interested in teasing out the drivers of binding (sequence or shape readout). When the data are too constraining, we should see the same binding mode regardless of the sequence. We compare multiple sequences for three systems involving different degrees of DNA bending upon binding (see Table 2).

**Table 2.** DNA sequences used for bZIP, TATA and P22 complexes

System	Sequence		PDB	Reference
**bZIP**	Consensus	CCTTGG**CTGACGTCAG**CCAAG	1DH3	(57)
	noCG	CCTTGGCTGA**AT**TCAGCCAAG		
	Random 1	CCTTGG**ATGCTACGAT**CCAAG		
	Random 2	CCTTGG**CGTAGCTCGG**CCAAG		
	Random 3	CCTTGG**TCTATCGGTT**CCAAG		
**TATA box**	Consensus	CTGC**TATAAAA**GGCTG	1CDW	(59)
	Domain 1	CTGC**CGCG**AAAGGCTG		
	Domain 2	CTGCTATA**GGG**GGCTG		
	Random 1	CTGC**CGCGGGG**GGCTG		
**P22 c2 repressor**	Consensus	CATTT**AAG**ATAT**CTT**AAATA	2R1J	(58)
	Domain 1	CATTT**CCT**ATATCTTAAATA		
	Domain 2	CATTTAAGATAT**GGC**AAATA		
	Bridge 1	CATTTAAG**CGCG**CTTAAATA		
	Bridge 2	CATTTAAG**CCGA**CTTAAATA		
	Random 1	C**GCCATTTAGGGACGATC**CA		

DNA bases highlighted in orange represent the changes with respect to the consensus sequence (bold).

### Protocol 3: competitive binding simulations quantify relative binding affinities

The data used in this protocol are the most constraining: we aim to distinguish the preference for a particular known binding mode to two different sequences. Thus, the data are compatible with one binding mode. Rather than defining this strictly as in docking or an alchemical free energy calculation, there is still enough freedom to sample widely inside the basin corresponding to this binding mode. To compare across systems, we have previously used a competitive binding strategy (65) in which the relative binding affinity can be determined by counting the population of the protein–DNA complex for each sequence. Converging populations to obtain statistical significance makes this protocol more computationally demanding than the previous two. We thus exemplify this protocol on the six sequences binding P22 shown in Table 2.

### HADDOCK docking predictions

We used the bio3d R module to calculate normal modes of the minimized canonical B-DNA and minimized protein for each system (66,67). The ensemble of normal modes for each binding partner was fed into the HADDOCK web server (68,69), specifying identical residues involved in the contact lists of MELD as active residues. We then analyzed the top five clusters and all the models generated by the docking package.

### RosettaFold2NA machine learning predictions

The RosettaFold2NA (RF2NA) program was assembled following their GitHub walkthrough (25). The FASTA sequence of each protein chain and DNA strand was provided to the program as separate files according to the instructions. Each prediction returned one structure.

## RESULTS

### MELD-DNA is successful at predicting protein–DNA complexes

**Figure 3.**
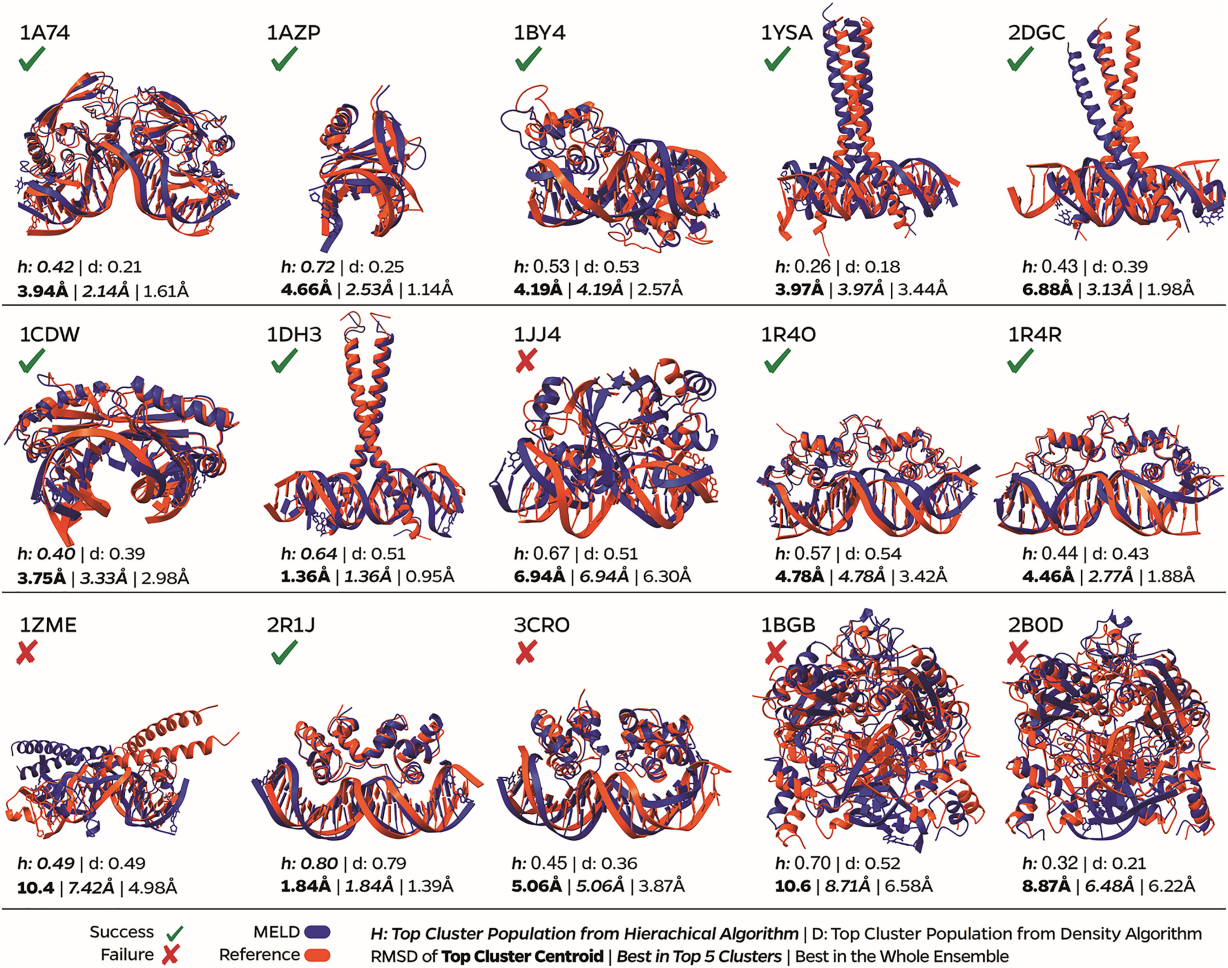
Superposition of the best in top five clusters of MELD simulations against the experimental structure. We report the interface RMSD of the top cluster, the best cluster amongst the top five clusters and the best structure in the ensemble. A prediction is marked as a success if we can find a <5 Å conformation in the top five clusters.

We analyze our ensembles for each of the 15 proteins by asking two questions (see Figure 3): (i) can the method sample the native state and (ii) can we identify the native state with high confidence without knowing the actual structure? We find that in 13 of the 15 cases the native state is present in the ensemble, and in 11 of 15 cases it belongs to a high population cluster (present in the top five clusters by population). The ensembles represent multiple binding modes, highlighting MELD’s ability to explore various bound conformations. Supplementary Figures S1–15 represent the distribution of structures sampled at every replica in the MELD approach for each of the 15 complexes. At high replica indexes, the system explores unbound conformations [high root mean square deviation (RMSD) values]. Sampling at lower replicas explores different bound states—where typically the lowest replica index will be enriched on the state with the lowest RMSD to the experimental structure. Complex 1AZP (see Supplementary Figures S2 and S16) represents a particular case since the DNA sequence is palindromic, and our methodology captures clusters binding in either orientation (180° flip of the protein; see Supplementary Figure S16). Similarly, most leucine zippers correctly identify the binding mode, with the binding domains overlapping the experimental binding mode. For these long coiled-coil structures, small fluctuations near the binding site give rise to a large displacement at the ends—hence some of these structures look visually distinct from the experimental structure but retain a low interface RMSD (e.g. 2GDG in Figure 3). Despite the use of restraints to keep the protein and DNA from unfolding, we find that both macromolecules sample a broad ensemble of conformations. Typically, the DNA and protein ensembles approach the holo conformation as the native binding mode is sampled (see Supplementary Figure S17).

We examined predictions from HADDOCK and RF2NA for comparison. For HADDOCK, we first tried submitting only one DNA and protein structure as input for each complex. As expected, in this scenario, we did not observe any native-like predicted complexes (which MELD also fails to predict in the absence of data). We then used several structures for both the protein and DNA sampled along their normal modes as calculated by the bio3D package (66,67,70). HADDOCK returns 200 models of the complex by default and clusters them. We observed many native-like complexes amongst the 200 generated models (Table 3; Supplementary Figures S18 and S19). However, the top five clusters generally had higher interface RMSDs and a lower fraction of native contacts (71,72) than MELD predictions. In our hands, the RF2NA machine learning approach produced overlaps between the two monomers in protein dimers where both bind in the same DNA region (marked as MO in Supplementary Table S1). For 1BY4, it used the two monomers of the protein to form a large monomer-like protein binding only on one of the two binding sites of the DNA. We are optimistic that future versions will be available to predict structures of homodimers binding DNA.

**Table 3.** Fraction of native contacts for MELD and HADDOCK (italics) from the top five clusters and the highest value sampled in the whole ensemble

		Fraction of native contacts					
System	No. of contacts	Cluster 1	Cluster 2	Cluster 3	Cluster 4	Cluster 5	Best in all
1A74	189	0.60	**0.85**	0.11	0.51	0.577	**0.93**
		*0.06*	*0.06*	*0.05*	*0.05*	*0.0*	*0.57*
1AZP	46	0.35	0.31	0.29	0.21	0.68	**0.95**
		*0.13*	*0.14*	*0.15*	*0.08*	*0.13*	*0.67*
1BY4	83	**0.73**	**0.74**	0.24	0.26	0.16	**0.80**
		*0.01*	*0.03*	*0.05*	*0.02*	*0.02*	*0.67*
1JJ4	109	0.15	0.31	0.06	0.00	0.02	0.34
		*0.33*	*0.41*	*0.23*	*0.28*	*0.12*	*0.58*
3CRO	123	0.38	0.10	0.13	0.08	0.16	0.57
		*0.06*	*0.4*	*0.04*	*0.06*	*0.1*	*0.62*
1ZME	110	0.17	0.27	0.09	0.13	0.15	0.62
		*0.02*	*0.06*	*0.04*	*0.06*	*0.01*	*0.49*
1DH3	55	**0.94**	0.04	0.11	0.34	0.49	**1.00**
		*0.32*	*0.38*	*0.40*	*0.24*	*0.27*	*0.44*
2R1J	99	**0.89**	0.18	0.30	0.31	0.10	**0.92**
		*0.05*	*0.03*	*0.04*	*0.02*	*0.05*	*0.62*
1CDW	84	0.51	0.20	0.07	0.18	0.49	0.69
		*0.08*	*0.12*	*0.04*	*0.04*	*0.07*	*0.58*
1YSA	62	0.64	0.58	0.36	0.67	0.52	**0.73**
		*0.38*	*0.44*	*0.26*	*0.22*	*0.05*	*0.51*
2DGC	72	0.50	0.62	**0.89**	0.47	0.68	**0.98**
		*0.04*	*0.04*	*0.05*	*0.03*	*0.21*	*0.36*
1R4R	83	0.50	0.27	**0.80**	0.29	0.42	**0.86**
		*0.01*	*0.01*	*0.01*	*0.01*	*0.02*	*0.26*
1R4O	79	**0.79**	0.45	0.34	0.34	0.31	0.86
		*0.02*	*0.04*	*0.02*	*0.03*	*0.03*	*0.69*
1BGB	220	0.09	0.08	0.02	0.05	0.12	0.19
		*0.00*	*0.00*	*0.00*	*0.00*	*0.01*	*0.10*
2B0D	227	0.20	0.17	0.07	0.17	0.19	0.24
		*0.00*	*0.00*	*0.00*	*0.00*	*0.01*	*0.18*

Any two residues with at least one heavy atom pair within 5 Å in the experimental structure were defined as a contact. Instances where ≥70% of the native contacts are satisfied are highlight in bold

### MELD is sensitive to sequence properties

**Figure 4.**
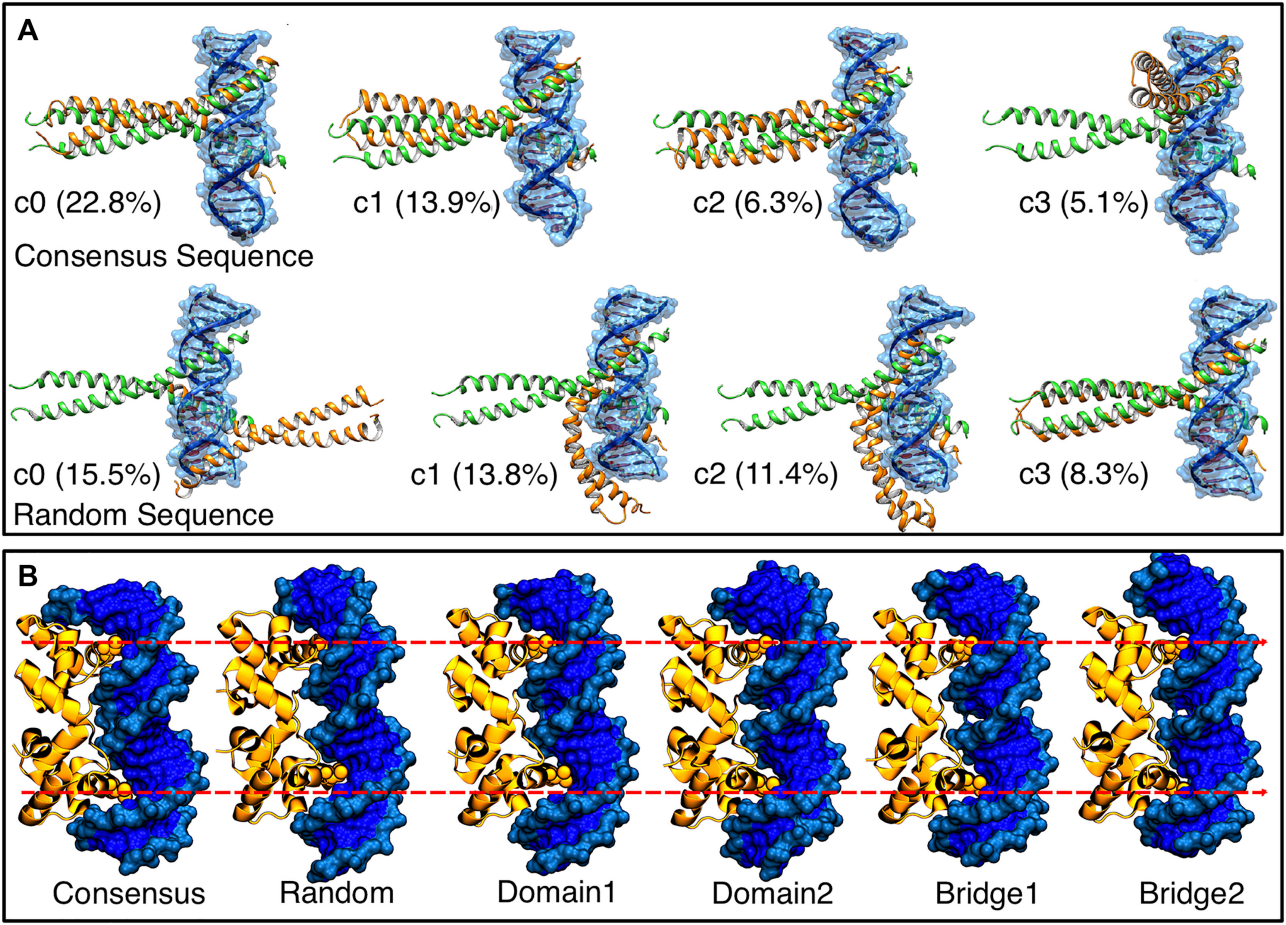
DNA sequence differences drive preferences in binding patterns. (**A**) Bzip binding patterns seen in the top four clusters (c0–c3) binding to either the consensus or a random sequence, ordered by population (in %). The plot shows multiple binding modes, with the consensus having a high population (22.8%) of the experimental binding mode compared with 8.3% for a random sequence. (**B**) Shape complementarity leads to similar binding modes across different sequences. Despite this, there are subtle differences in the number of base pairs between the two binding sites (denoted by the red dotted lines).

We chose three systems representative of binding DNA in its canonical conformation, bent DNA and significantly bent DNA (bZIP, P22 and TATA box-binding proteins, respectively). For each of the systems, we simulated the consensus sequence, for which we have an experimental structure of the complex. We then generated the sequences defined in Table 2, for which we have no experimental data. The consensus sequence is the highest affinity binder of all sequences. However, other sequences are also likely to bind based on charge complementarity—especially in cases with no specific interactions between protein residues and DNA nucleobases. Hence, we expect differences in the ensemble, either by binding at different sites along the sequence or by affecting the populations identified by clustering with respect to the consensus sequence (see Figure 4).

For all three cases, MELD correctly identifies the native binding mode when the consensus sequence is used; however, we differentiate two different behaviors when changing the sequence. For P22 and TATA box-binding protein, binding is driven by shape complementarity, and, in all simulations, we observe a very similar binding pattern, with all DNA sequences adapting bent conformations. However, for bZIP, which binds DNA in its canonical B-DNA conformation, we observe dramatic shifts in cluster populations and binding preferences, with some sequences preferring to bind different sites along the sequence (see Figure 4A). To further study the sequence/structure relationship, we took the top two clusters from unbiased molecular dynamics simulations of each protein-free DNA sequence, and we performed binding simulations restraining the conformation of each sequence to each of the possible clusters (a total of 50 simulations). Supplementary Figure S20 shows the RMSD of the protein versus the protein–DNA interface RMSD. While some structure/sequence combinations lead to binding, others do not. For the consensus sequence, the experimental binding mode is sampled in high populations in 9/10 simulations, with the highest population cluster falling in this region most of the time. Other sequences have a lower preference for the canonical binding site despite starting from the same conformation and using the same data. Conformation random1-c1 (random1 sequence and cluster 1 conformation) merits a special mention: only using the sequence from random1 does this conformation allow sampling of the experimental binding mode. For this structure, we find a significant amount of binding through the minor groove in most sequences; however, for Random1 sequence, the most prevalent binding mode is through the major groove, as in the experimental structure. Thus, we conclude that the physical model used in MELD is sensitive to sequence-dependent properties. Despite this, in cases where the DNA undergoes significant conformational changes, where we expect shape readout to drive binding, the MELD restraints overcome sequence preferences. It is difficult to directly compare the effect of the restraints as independent MELD binding simulations are not comparable (73). We thus complement these results with relative binding affinity calculations (protocol 3).

### Relative binding affinities identify structure/sequence preferences during binding

Here two DNA duplexes and a protein are simulated together (see the Materials and Methods), promoting protein binding to either DNA duplex at low replicas while restricting interactions between the duplexes. During unbinding events, the protein is far from both DNA duplexes. We assess the higher affinity binders by counting how often the protein binds each duplex. In this approach, the binding mode is known; thus, MELD uses more constraining information to favor faster binding/unbinding events, leading to higher statistical significance.

**Figure 5.**
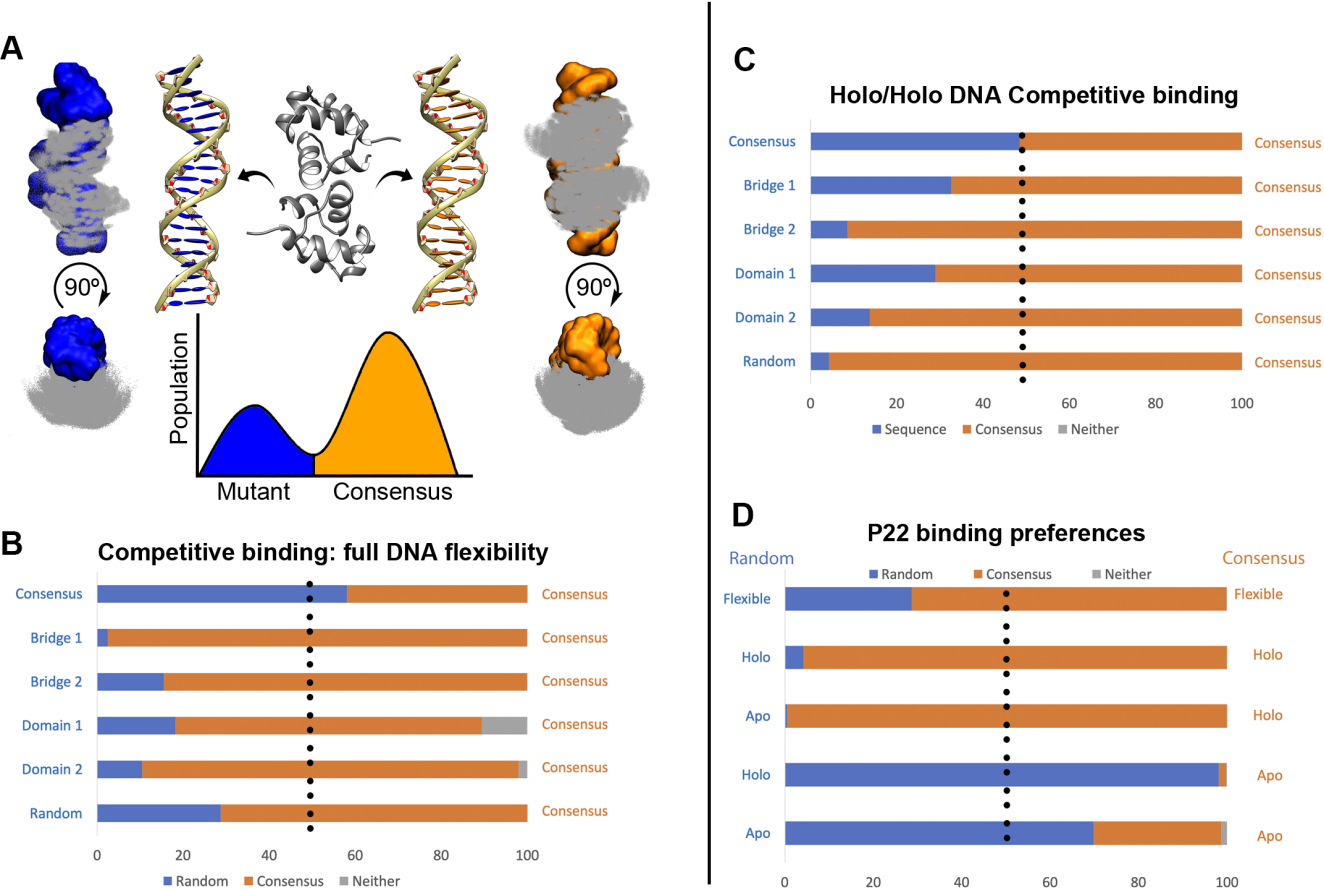
P22–DNA competitive binding. (**A**) MELD simulations in which the protein is directed to bind to two different DNA sequences (blue and orange). Protein is shown as Cα dots superposing all frames binding to either sequence. The ratio of populations is related to the relative binding affinity. (**B**) When the DNA is allowed to change its conformation during competitive binding freely, the consensus sequence has a marked preference for binding over the mutant (population in each mode in the bar, with gray depicting non-native binding modes). (**C**) Restraining DNA conformations to their holo conformation removes the conformational free energy to adopt bound conformations. Despite this, the consensus sequence remains the most likely to bind. (**D**) Comparison of random and consensus sequences allowing complete DNA flexibility or restraining either sequence to the holo/apo conformations.

We first tested simulations in which we competed the consensus sequence against itself. The expected outcome is that the protein should bind equally to each sequence and is thus a test of the expected errors as well as possible systematic errors arising from the set-up conditions. The observed ratio of 57/43 populations was close to the expected 50/50 value (see Figure 5B), giving us confidence that the set-up conditions were not biased towards one of the two DNA structures. Competing the consensus against the other five sequences showed that the consensus sequence was preferred in all cases.

The advantage of simulation tools is that we can decouple sequence and shape readout by freezing the DNA structure in either its apo or holo conformation—similar to a rigid docking experiment, where the DNA can only fluctuate around the initial structure. We expected all relative free energies to increase when freezing DNA structures to the holo conformation as the conformational free energy change to the binding free energy was ‘pre-paid.’ Interestingly, while it increased in most cases (see Figure 5D), it decreased in others, most significantly for the random sequence. We further analyzed this sequence by fixing either the consensus or random sequence to the holo/apo conformation. Surprisingly, the protein recognizes the random sequence preferentially when both structures are kept in their B-DNA form but the consensus sequence when they are in their holo conformation. Thus, simulations that include flexibility to deform will have more events progressing down the replica ladder when the protein binds the random sequence. Still, once they form the complex structure, the consensus remains bound for a longer time. The approach is not currently sensitive to large free energy differences—where only one structure might be bound at the lowest replica. The method can readily be made more quantitative by using information from all replicas with proper reweighting [e.g. using the multistate Bennett acceptance ratio (74)]; however, this was beyond the scope of the current work (74).

## DISCUSSION

The MELD approach is designed to help answer questions involving structural and energetic considerations, drawing from DNA’s sequence dependence binding preferences. Choosing the origin of the information can help in either refining structures from these methods (e.g. making predictions from these methods and generating ambiguous/noisy data to guide MELD binding), answering questions about shape/sequence readout or even relative binding affinities. The general pipeline is available on GitHub (github.com/PDNALab/MELD-DNA) to showcase the potential of this framework. Overall, the approach successfully predicts the structures of protein–DNA complexes. Understanding sequence/structure relationships and relative binding free energies is better indicated for systems where existing available information reduces the conformational search space. The trade-off between efficient sampling and the physics model to use currently limits MELD-DNA to implicit solvent, which is known to be less accurate than simulations in explicit solvent. Nonetheless, the method samples accurate atomic structures representative of the native state for most of the systems studied, even when starting from B-DNA conformations. The physics-based nature of the ensembles readily makes this a valuable approach to obtaining structures for more detailed studies (e.g. in explicit solvent), such as using different MELD clusters as seeds for adaptive sampling simulations combined with Markov state models (75,76). Thus, we believe the current framework can be a powerful tool to increase our understanding of nucleic acid complexes.

We have shown a successful strategy for identifying protein–DNA complexes sensitive to the DNA sequence, exploring multiple binding modes and which samples DNA deformation during binding. The MELD approach draws on the successes of the HADDOCK docking strategy of combining ambiguous and noisy data with the search engine. It goes beyond harmonic deformations by using a molecular dynamics engine sensitive to the DNA sequence and identifying successful structures based on a statistical mechanics treatment of the generated ensemble rather than on scoring functions. It is worth noting that we employed HADDOCK as regular users versed in structural biology, and expert users might improve the performance. Regardless, while the accuracy in HADDOCK structure predictions is lower than that of MELD, it also comes at a small fraction of the computational cost: MELD simulations require 30 GPUs typically running for about 2 days for these systems, while HADDOCK calculations take a few minutes, on a single core. HADDOCK predictions can readily be incorporated as structural seeds for each replica in MELD, as well as a source of ambiguous/noisy information. In this regime, MELD can be used to refine HADDOCK’s models while simultaneously identifying the most likely model as the one most prevalent in the lowest temperature ensemble (77).

The choice of restraints, noise and ambiguity in the dataset will depend on how much data are available to the user. Higher accuracy and amount of data lead to faster convergence but reduced exploration of the binding landscape. For most purposes of structure prediction, protocol 1 is the most transferable and generalizable. Furthermore, users might decide to change the restraints imposed on the protein and DNA. In our current approach, the DNA has more flexibility than the protein system (see Supplementary Figure S17) to account for DNA bending during binding. However, restraints between base pairs will keep structures from sampling conformations where one base is open (e.g. for binding methyltransferases). For such cases, a user would modify the restraints affecting the desired region of DNA. Similarly, the protein restraints affect the ability to sample open/closed states, which are needed for some systems where the binding site is not accessible in the closed state. Knowing the binding site, exceptions can be made on which regions of the protein to restrain.

We see three issues related to (i) accessibility of the binding site, (ii) force field accuracy and (ii) efficiency of replica exchanges. Proteins 1BGB and 2B0D require an opening event before the DNA can access the binding site; however, the standard protocol used to predict binding (protocol 1) uses distance restraints that prevent the protein from unfolding and, in this case, from accessing the open conformation state. Furthermore, force fields typically have a bias towards compact structures, especially in implicit solvent, further favoring compact closed conformations that prevent DNA binding. Some authors have also suggested a possible DNA + protein force field imbalance resulting in stronger than expected arginine and lysine interactions with the phosphates (78–81). Such highly charged systems challenge the accuracy of the force field in the implicit model. This issue is best seen in our set of complexes for the TATA box-binding protein (1CDW). The bend in DNA structure induced by binding of the TATA box-binding protein is further accentuated in the implicit solvent (see Supplementary Figure S21), resulting in overly strong electrostatic interactions (see “Specific Results" in the Supplementary Information). Because of compaction and these strong electrostatic interactions, replica exchanges that favor unbinding have a lower probability than similar approaches for protein–protein and protein–peptide binding (see Supplementary SI; Supplementary Figures S22 and S23). Thus, despite the current success, future endeavors will aim to introduce explicit solvent into the methodology.

Overall, the MELD-DNA methodology we presented herein fills a gap in computational tools that predict protein–DNA binding. We have shown that the method is sensitive to sequence and structural preferences and is thus a promising new approach to studying this type of system. The MELD code is freely available through GitHub as a plugin to openmm (82). On a diverse set of protein–DNA systems involving 15 different complexes, the method successfully predicted 10 of them as high population clusters. We believe the physics-based insights which MELD-DNA can provide will advance our understanding of protein–DNA interactions and our ability to simulate events related to supramolecular chemistry. Increasing our structural knowledge and sequence binding structural preferences combined with other factors that affect *in vivo* binding (e.g. chromatin state and accessibility) can bring new understanding to the molecular mechanisms that orchestrate gene regulation.

## DATA AVAILABILITY

The MELD software is distributed freely and is available through the GitHub repository (github.com/maccallumlab/meld); a permanent Zenodo link is accessible at https://doi.org/10.5281/zenodo.7502226. Scripts and sample data used for this report are available at our group's GitHub (github.com/PDNALab/MELD-DNA), and a permanent copy has been deposited on Zenodo (https://doi.org/10.5281/zenodo.7501938).

## Supplementary Material

gkad013_Supplemental_File
