# Supplementary material for: Structural predictions of protein–DNA binding: MELD-DNA
Source: Nucleic Acids Res. 2023 Feb 2;51(4):1625–36. doi: 10.1093/nar/gkad013 (PMC9976882; doi:10.1093/nar/gkad013)
Supplement: gkad013_Supplemental_File [file gkad013_supplemental_file.pdf]

SI METHODS

Explicit solvent simulations of “free” DNA

For each DNA sequence in Table 1 we performed Langevin dynamics simulations in explicit solvent using the TIP3P(1) water model, parmbsc1(2) and 150mM KCl(3), running for 500ns. Initial conformations were generated based on ideal B-DNA helical parameters using the nucleic acid builder(4). We clustered the last half of the ensemble, clustered using a hierarchical agglomerative method (ε=2) and retained the top cluster centroids as starting points for binding simulations.

Binding simulations with MELD-DNA

Protein-DNA recognition using MD based approaches is hampered by the large conformational landscape to explore. The Modeling Employing Limited Data (MELDxMD(5)) approach uses ambiguous and noisy information to reduce the conformational landscape and an MD engine to provide a physical model. Both approaches are integrated through Bayesian inference, using the physics model as the *prior* (p(x)) distribution and the *likelihood* (p(D|x)) represents *the best interpretation* of the data given the current structure (see Fig. 1). A key point here is that MELD allows for noise and ambiguity in the dataset. Thus, rather than using all the data simultaneously to guide sampling, only a subset of the data is enforced at any point. At each step all the data is evaluated, and the subset with greater agreement with the current structure is used until the next step, where the calculation is repeated. In this way, no information is ever discarded. For efficient sampling of multiple binding/unbinding events, the protocol uses a Hamiltonian and Temperature Replica Exchange approach (H,T-REMD)(6). The resulting ensembles are analysed by hierarchical clustering using a similarity measure (RMSD) and an ε of 2 is performed to identify the most likely conformations (the cluster with the highest population)(7). For more details about the methodology and properties of the ensemble we direct readers to the method papers(5, 8).

MELD uses H,T-REMD for enhanced sampling

The MD engine generates new structures according to Langevin dynamics, using the parmBSC1 force field for nucleic acids(2, 9, 10) the ff14SB side force field for the protein(11, 12) and the GBneck2Nu implicit solvent model(13, 14). Each simulation ran for at least 1μs using a 4.5fs timestep and hydrogen mass repartitioning. How much data is to be trusted (the size of the subset) is given by an accuracy parameter that is chosen based on the type of data. In this work the restraints were modelled as flat-bottom harmonic restraints between pairs of atoms in the protein-DNA system, where one atom comes from the protein and one from the DNA. The Hamiltonian changed between replicas by scaling the force constant of the restraints. We use 30 replicas in all simulations and create functions that map each replica to a temperature and Hamiltonian. To make the process generalizable, we map each replica to a number between 0 and 1 (alpha, α). We use a geometric function of alpha for the temperature and a non-linear function to map the Hamiltonian(15, 16). At α=1, the system is in full exploration, running at 500K and with no biasing restraints. At α=0, the system is in exploitation mode, sampling at 300K and with strongly enforced biases (k=250

kJ/nm^2). Effectively, the REMD approach at high temperatures allows jumping between different regions of the conformational landscape and the low replica indexes exploit those minima.

Data used in binding simulations

Increasing the temperature in the replica exchange ladder rapidly leads to DNA and protein denaturalization. To favor binding/unbinding without denaturing these molecules we impose internal restraints.

Protein restraints: we calculate internal C_α-C_α distances based on the initial experimental structure and impose them through-out the trajectory to limit unfolding. All C_α-C_α distances below 8Å in the crystal structure are selected and enforced using flat-bottom harmonic restraints with a flat-bottom region expanding 1Å in each direction of the calculated distances, to allow higher flexibility. We further increase system flexibility by requiring that only 95% of the restraints be satisfied at any given time.

DNA restraints: we impose hydrogen bond restraints across Watson-Crick pairs to prevent the two strands from separating. These restraints allow DNA bending and is thus suitable to sample bound conformations in the presence of the protein, starting from a B-DNA structure.

Protein-DNA restraints guide binding along multiple binding modes

We use multiple pair-wise restraints between the protein and DNA to guide them towards each other. Different subsets of restraints are compatible with different binding modes, which are sampled through the MELD approach. We present three protocols in the main text to address different types of scenarios a molecular modeler might encounter. However, the data can also originate from experiments, or be specifically designed based on previous knowledge of the system under study.

Decoupling binding from shape readout

In certain types of simulations, we ask whether binding would occur if the DNA was in the bound (our unbound) conformation. This aims to address questions of *shape* recognition and are especially useful during competitive binding simulations (see below). In these cases, we restrain the DNA to an initial conformation imposing flat-bottom harmonics cartesian restraints on all heavy atoms. For each atom, deviations within 3.5Å of the initial position are allowed with no penalty. Beyond this distance, a harmonic potential with k=250 kJ/nm^2 limits the conformational ensemble available to DNA.

Competitive binding simulations

MELD-DNA binding simulations identify preferred binding conformations but does not provide information about the binding free energy. Competitive binding simulations allows us to recover relative binding free energies(15, 16). The data is compatible with either DNA sequence with equal probability, requiring that when the protein is interacting with one DNA sequence, the other sequence is far away in a reference state. The reference state is chosen to be the same for each sequence in such a way that counting populations of binding to each sequence at the lowest replica provides a relative binding free energy(15, 16)We have

previously shown that the MELD relative binding free energy is related to the binding free energy between the two structures(15). In competitive binding, the system consists of the protein and two DNA molecules. The two DNA structures are restrained far away from each other (between 50 and 100Å from each). These restraints prevent the protein from interacting with both DNA sequences simultaneously. Each restraint in this protocol can be satisfied between the protein and either DNA molecule. At high replica indices the protein is far away from both DNA structures, while at low replica indices the protein is bound to either DNA structure. The non-bound sequence remains in its reference state (at distances greater than 50Å) sampling mostly B-DNA conformations. Analyzing the lowest replica shows the protein "jumping" between the two DNA molecules. A simple ratio of the bound population to each DNA structure yields binding preferences (see Fig. 3).

These simulations aim to tell us about the ability of the protein to recognize sequence or shape and the ability of a particular DNA sequence to deform to the bound conformation. Thus, we restraint the DNA conformation using cartesian restraints as previously described. For the simulations in which the DNA is free to deform, we added a flat-bottom harmonic distance restraint to keep the distance between the two DNAs from coming close with each other or too far away from each other. The flat bottom region is set up to keep the two DNA sequences at a distance between 50 and 70 Å from each other.

Specific Results

Rational for sequences in protocol 2

Since binding affinities of TF proteins to many different sequences is expected to be high, and MELD drives simulations towards bound conformations, we expect that in all cases we will see clusters that are representative of the experimental binding mode. Note here that if MELD is exploring efficiently, the experimental binding mode will be reproduced along different sites in the protein (e.g., a displacement of where along the sequence the protein binds). In this view, we expect that a certain protein has an associated preferred binding mode – and the DNA sequence determines where along the sequence this binding mode results in a higher binding affinity.

Thus, to design sequences we looked at features in the binding site that grant specificity to each complex. In the case of bZIP this is the central CG step. Hence, we designed sequences that are expected to introduce large perturbations to the system. If our hypothesis is correct, we should see complex formation at a different site along the sequence. This should be reflected in changes in populations of the different clusters – we expect that the binding mode at the same site along the sequence will have a lower population that when the consensus sequence is used. Indeed, we observe this behaviour for the bZIP system (see Fig. 4A). For the P22 systems the effect is more subtle, where we see same binding site and mode, but differences in how many base pairs are in between the binding site of the two domains. On the other hand,

the TATA system shows an anomaly during biding in which the DNA is bent even beyond what is observed in the experimental structure. The stable conformations had a protein-DNA RMSD between 3.5Å and 4.5Å, with internal DNA conformations greater than 5Å. The protein loop-to-loop distance was reduced from 30.8Å in the experimental structure to 19.0Å in our most populated cluster. This in turn increases DNA kinking (see Figure S21). We observe differences in binding modes corresponding to small displacements of the binding mode along the DNA sequence.

Although the force field/implicit combination fails to capture the native state at the lowest replica for the TATA complex, we observe and enrichment of this state in replicas 11-20 – which correspond to higher temperature conditions (see Fig. S22). We tested the ability to sample and identify correct binding poses as well as identify sequence preferences by restraining DNA conformations close to its HOLO conformation. The consensus DNA sequence contains a TATAAAA sequence at the binding interface, which maps to an interaction with each structural repeat in the protein for four different sequences. All four simulations identify the bound conformation, with protein-DNA RMSDs close to 2.5Å as the most populated cluster at low population, and the internal conformation of DNA deviating between 2 and 3Å from the starting conformation (see Fig. S23).

Competitive binding simulations for P22

The free energy of binding has contributions from the change in conformation in the protein, DNA and from their interaction (see Equation S1).

$$\Delta G_{bind} = \Delta G_{protein} + \Delta G_{DNA} + \Delta G_{interaction} \quad \text{(Equation S1)}$$

The free energy contribution for the protein deformation is similar when binding different DNA sequences. However, the free energy for deforming DNA to their bound conformations changes significantly according to sequence dependent properties (28-30). The interaction free energy will depend mostly on whether the interaction takes place through specific sites present on only some sequences or through general features (e.g. DNA backbone). By restricting conformations of the DNA to either their apo/holo conformation or allowing full flexibility to the DNA the simulations can discern the role that shape/sequence recognition play in the binding process.

We compete pairs of sequences, running four experiments for each pair where the DNA is restricted to a region of conformational space (either bound or unbound) and one experiment in which the DNA is allowed to freely deform. For the restrained simulations we perform four experiments, including:

(i) Apo (unbound conformation) *vs* Apo sequence
(ii) Holo (bound conformation) *vs* Holo sequence
(iii) Holo *vs* Apo sequence

These simulations aim to tell us about the ability of the protein to recognize sequence or shape and the ability of a particular DNA sequence to deform to the bound conformation. For the simulations in which the DNA is free to deform, we added a flat-bottom harmonic distance restraint to keep the distance between the two DNAs from coming close with each other or too far away from each other. The blat bottom region is set up to keep the two DNA sequences at a distance between 50 and 70 Å from each other.

System	MELD			HADDOCK			RF2NA
	Top Cluster	Top 5	Ensemble	Top Cluster	Top 5	Ensemble	Model 0
1A74	**3.94**	2.14	1.61	10.78	19.84	3.34	18.2
1AZP	4.66	**2.53**	1.14	5.96	5.96	1.85	7.3
1BY4	4.19	4.19	**2.57**	13.61	13.1	3.70	N/A
1JJ4	6.94	6.94	6.30	9.56	9.14	7.98	7.9
3CRO	5.06	5.06	**3.87**	9.59	7.98	7.79	MO
1ZME	10.4	7.42	4.98	10.76	10.3	2.4	12.9
1DH3	**1.36**	1.36	0.95	5.82	5.41	4.84	MO
2R1J	**1.84**	1.84	1.39	4.90	371	2.57	MO
1CDW	**3.75**	3.33	2.98	9.68	9.68	2.89	14.1
1YSA	**3.97**	3.97	3.44	21.65	20.9	8.44	5.8
2DGC	6.88	**3.13**	1.98	8.67	7.32	2.92	MO
1R4R	4.46	**2.77**	1.88	18.21	18.0	2.86	MO
1R4O	4.78	4.78	**3.42**	19.64	7.09	2.79	MO
1BGB	10.6	8.71	6.58	6.65	6.42	5.9	MO
2B0D	8.78	6.48	6.22	13.13	7.54	6.03	MO

Table S1. Interface RMSD of protein-DNA compared from MELD, HADDOCK and RF2NA. For MELD and HADDOCK, RMSD of top cluster, best in top 5 clusters and best in the whole ensemble is shown. For RF2NA, RMSDs were calculated using chimera from the interface residues used elsewhere in the paper. MO: Monomer Overlap. For 1BY4, we observed that the two monomers of the protein were used to make a large monomer-like protein binding to one side of the DNA. Bold numbers represent the level at which MELD simulations detect structures with an RMSD lower than 4Å (in 5 cases just based on the top cluster, three more systems can be successfully predicted by looking at the top 5 clusters, and for 3 more systems the ensembles sample native like complex structures that we cannot identify based on statistical mechanics). Thus – four systems are considered MELD sampling failures.

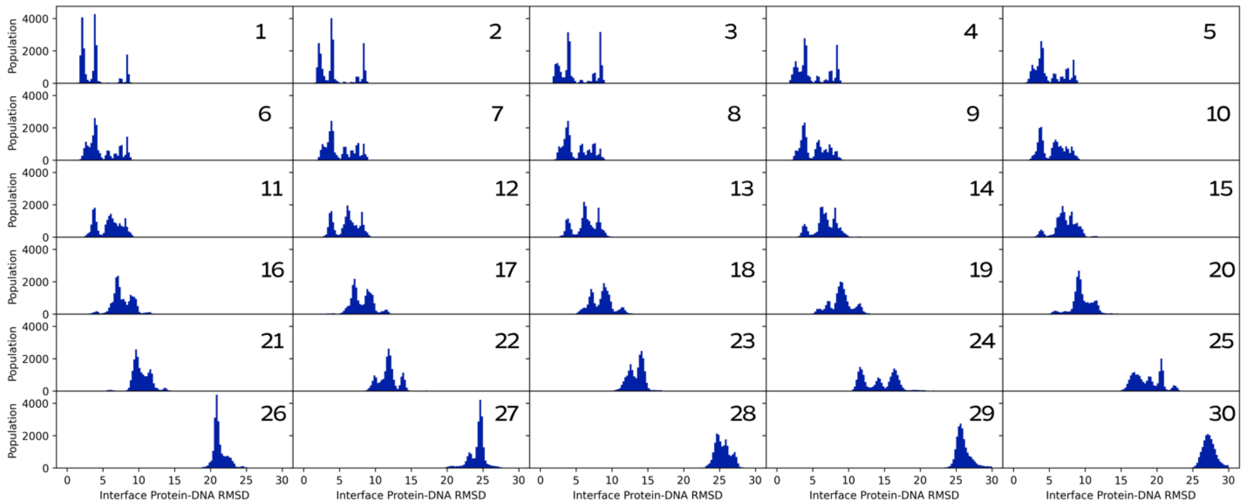

Figure S1. Histogram of ensemble RMSDs for 1A74. Each panel corresponds to a different replica conditions (indicated by the number in the panel). Replica index 1 explores bound states at the lowest temperature while replica 30 explores unbound states at the highest temperature.

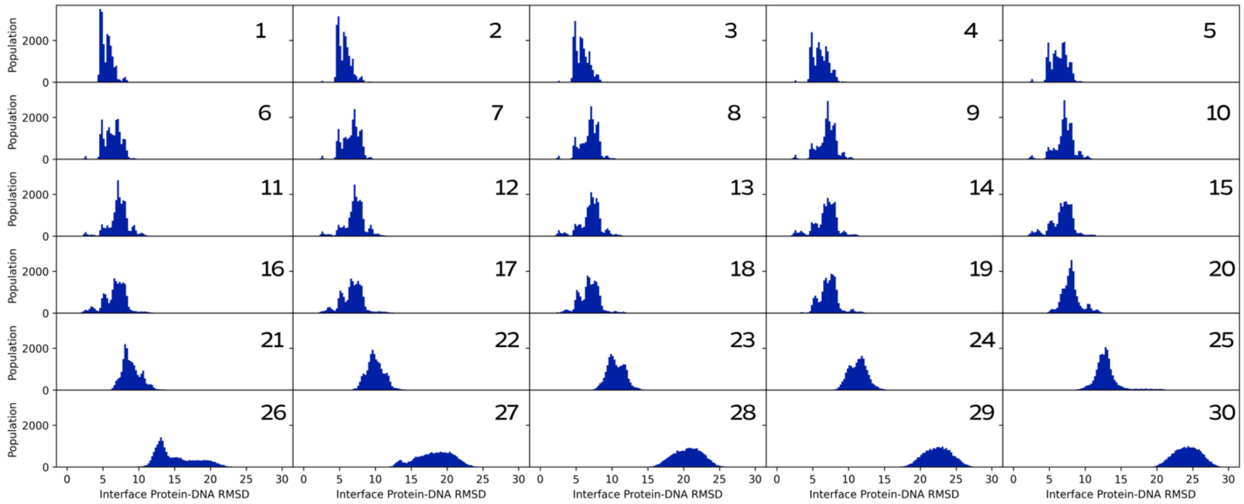

Figure S2. Histogram of ensemble RMSDs for 1AZP. Each panel corresponds to a different replica conditions (indicated by the number in the panel). Replica index 1 explores bound states at the lowest temperature while replica 30 explores unbound states at the highest temperature.

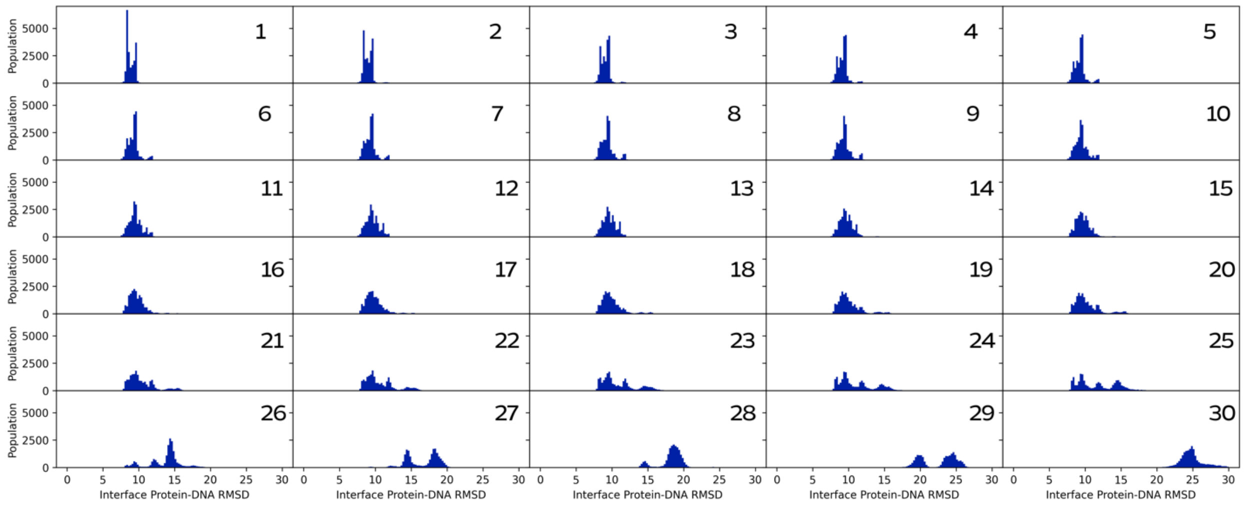

Figure S3. Histogram of ensemble RMSDs for 1BGB. Each panel corresponds to a different replica conditions (indicated by the number in the panel). Replica index 1 explores bound states at the lowest temperature while replica 30 explores unbound states at the highest temperature.

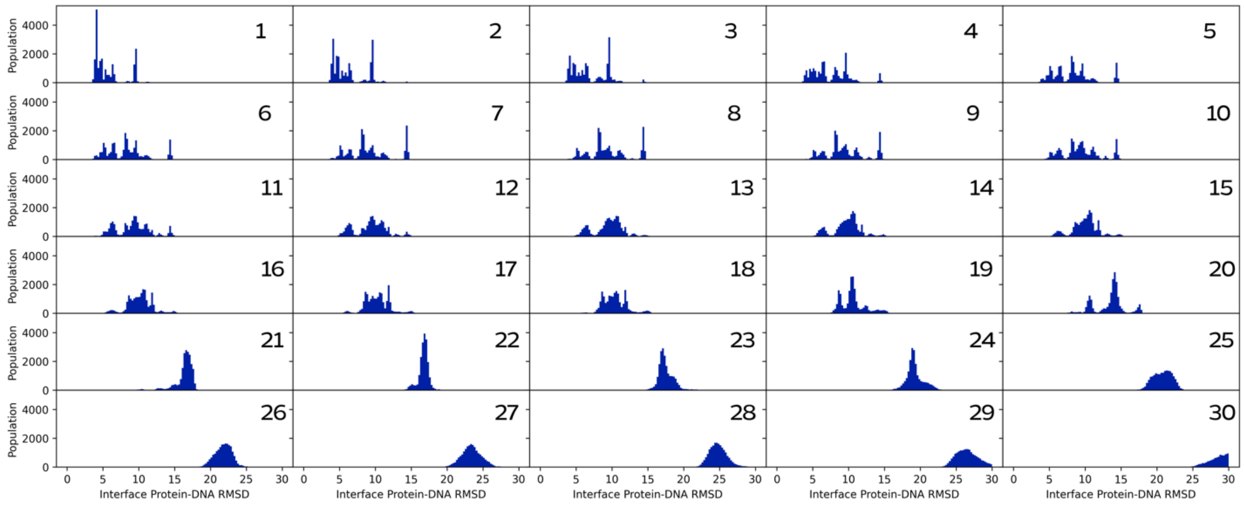

Figure S4. Histogram of ensemble RMSDs for 1BY4. Each panel corresponds to a different replica conditions (indicated by the number in the panel). Replica index 1 explores bound states at the lowest temperature while replica 30 explores unbound states at the highest temperature.

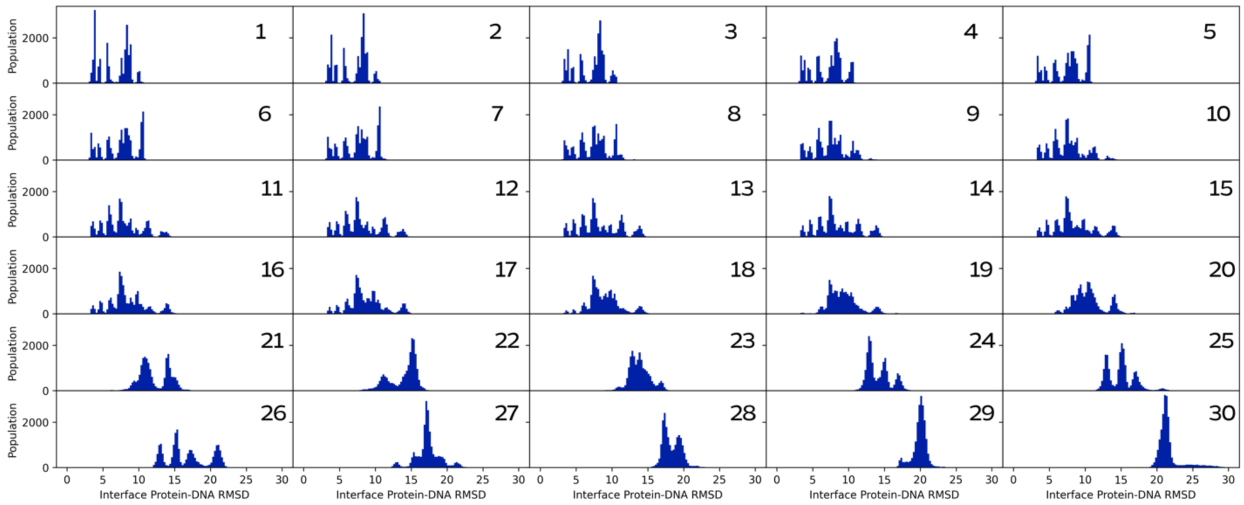

Figure S5. Histogram of ensemble RMSDs for 1CDW. Each panel corresponds to a different replica conditions (indicated by the number in the panel). Replica index 1 explores bound states at the lowest temperature while replica 30 explores unbound states at the highest temperature.

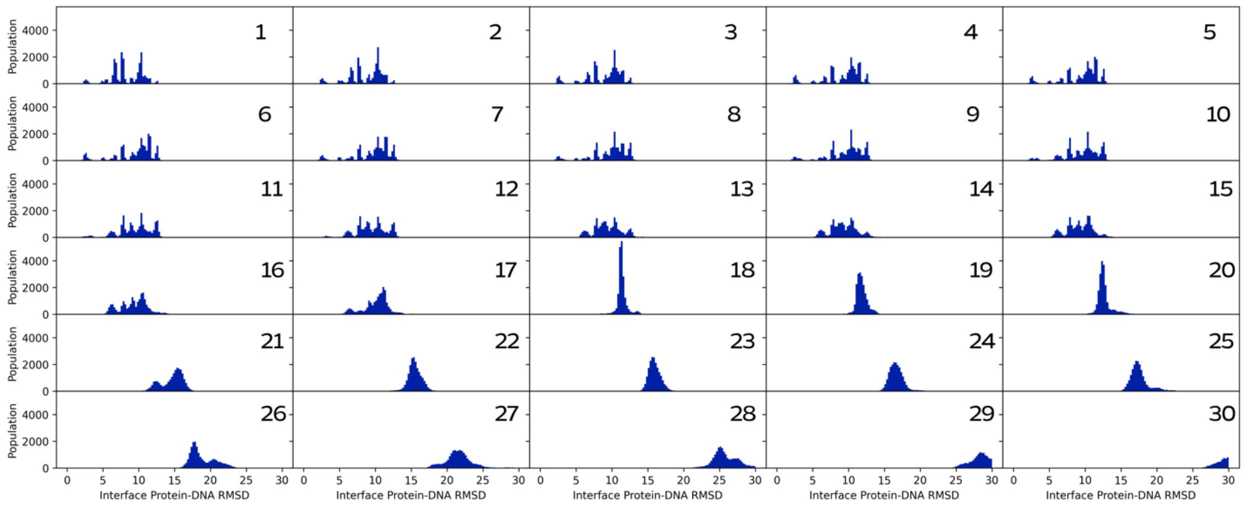

Figure S6. Histogram of ensemble RMSDs for 1DH3. Each panel corresponds to a different replica conditions (indicated by the number in the panel). Replica index 1 explores bound states at the lowest temperature while replica 30 explores unbound states at the highest temperature.

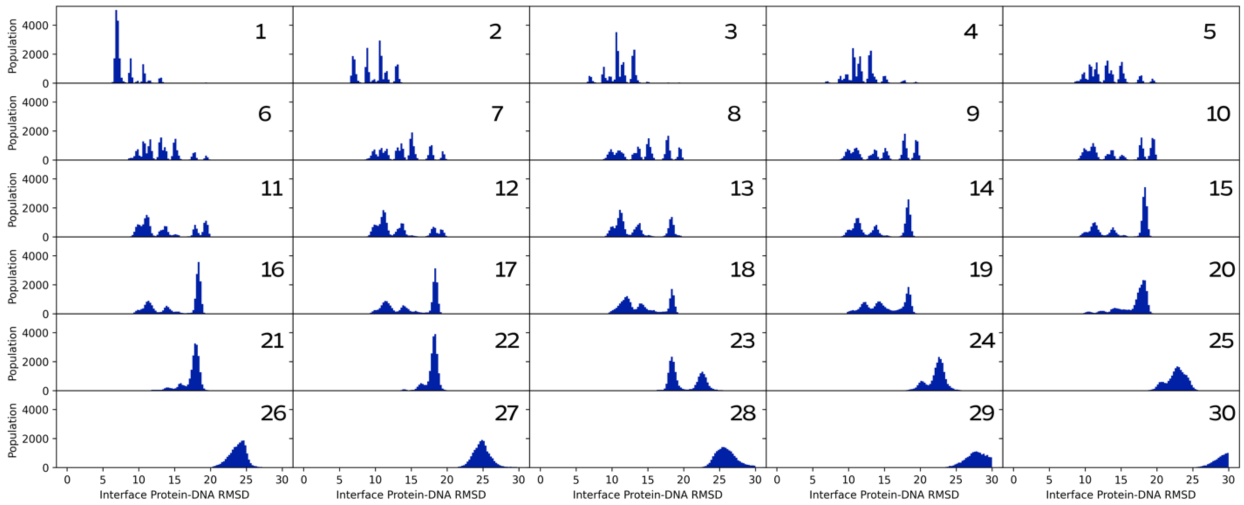

Figure S7. Histogram of ensemble RMSDs for 1JJ4. Each panel corresponds to a different replica conditions (indicated by the number in the panel). Replica index 1 explores bound states at the lowest temperature while replica 30 explores unbound states at the highest temperature.

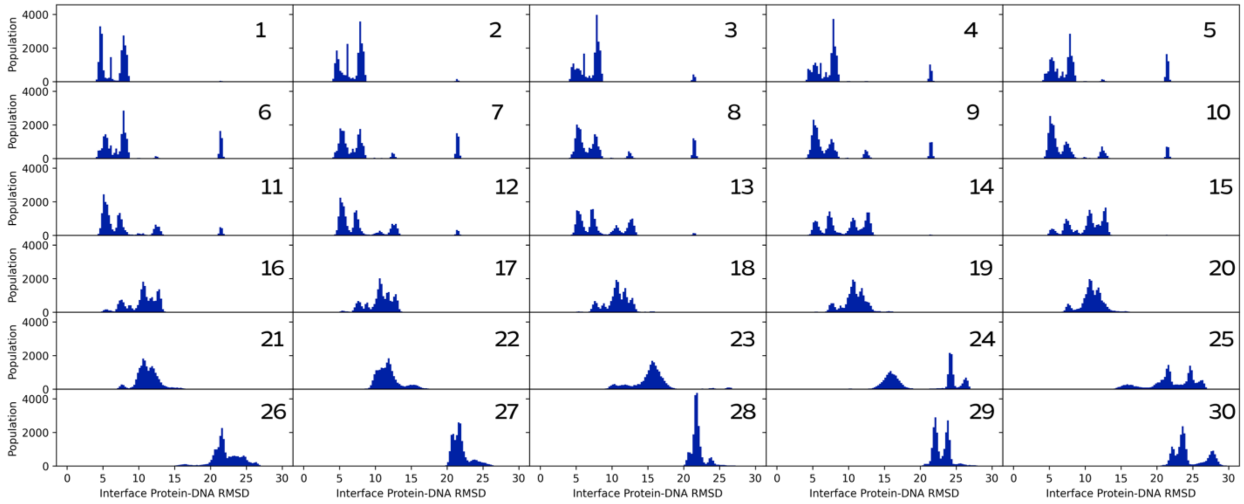

Figure S8. Histogram of ensemble RMSDs for 1R4O. Each panel corresponds to a different replica conditions (indicated by the number in the panel). Replica index 1 explores bound states at the lowest temperature while replica 30 explores unbound states at the highest temperature.

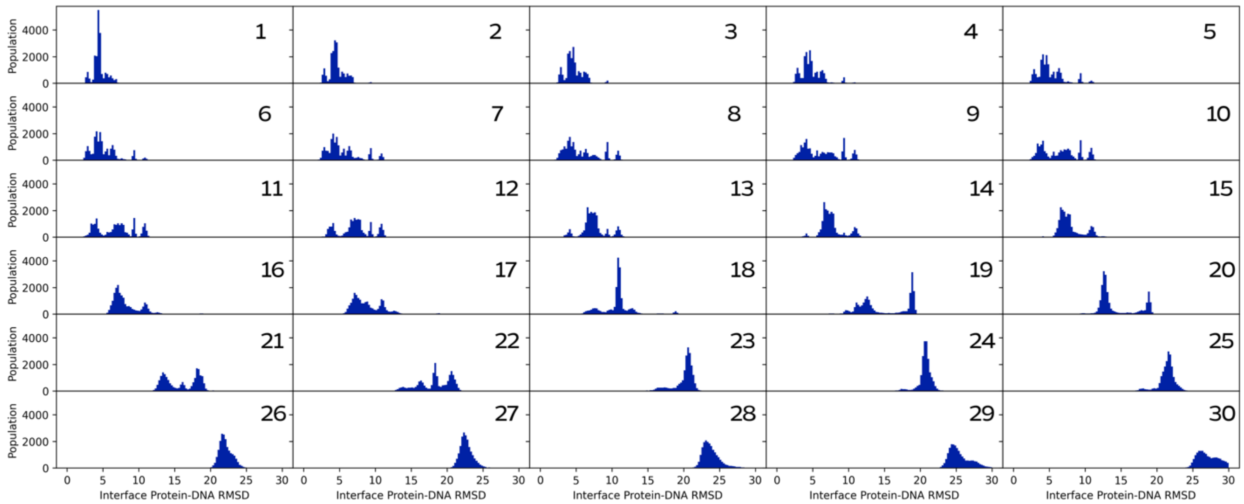

Figure S9. Histogram of ensemble RMSDs for 1R4R. Each panel corresponds to a different replica conditions (indicated by the number in the panel). Replica index 1 explores bound states at the lowest temperature while replica 30 explores unbound states at the highest temperature.

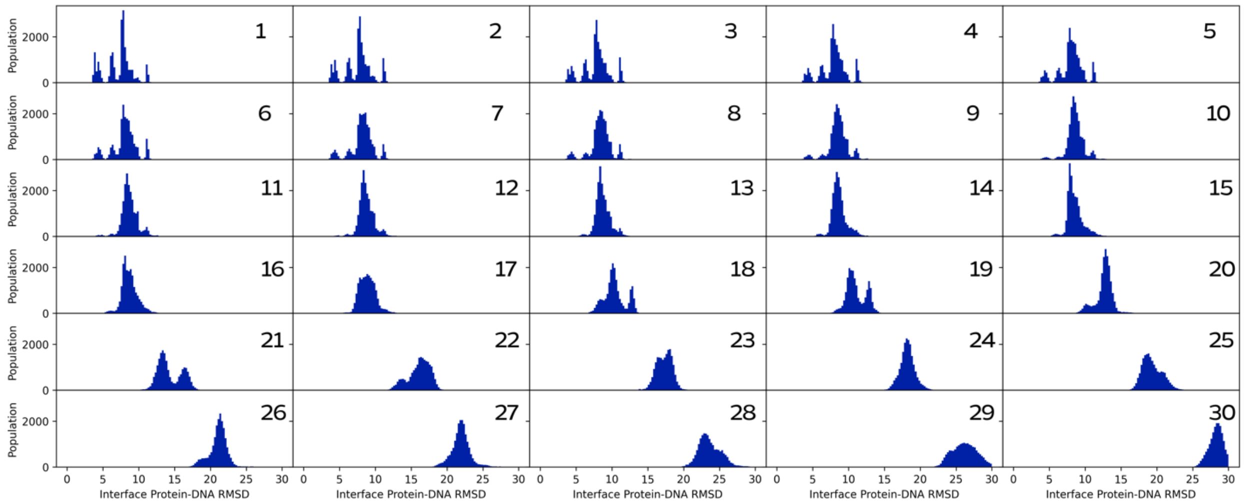

Figure S10. Histogram of ensemble RMSDs for 1YSA. Each panel corresponds to a different replica conditions (indicated by the number in the panel). Replica index 1 explores bound states at the lowest temperature while replica 30 explores unbound states at the highest temperature.

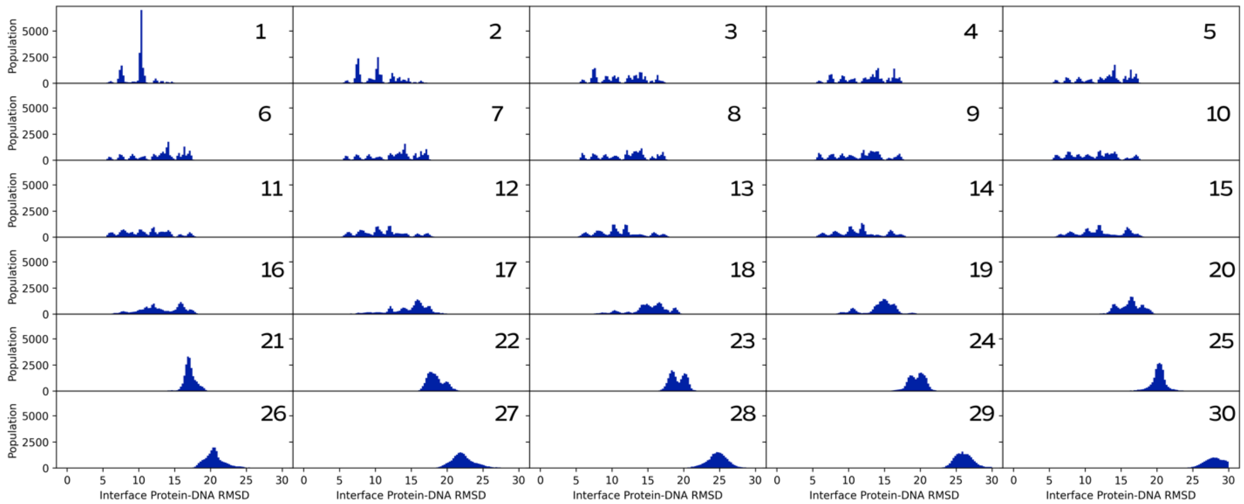

Figure S11. Histogram of ensemble RMSDs for 1ZME. Each panel corresponds to a different replica conditions (indicated by the number in the panel). Replica index 1 explores bound states at the lowest temperature while replica 30 explores unbound states at the highest temperature.

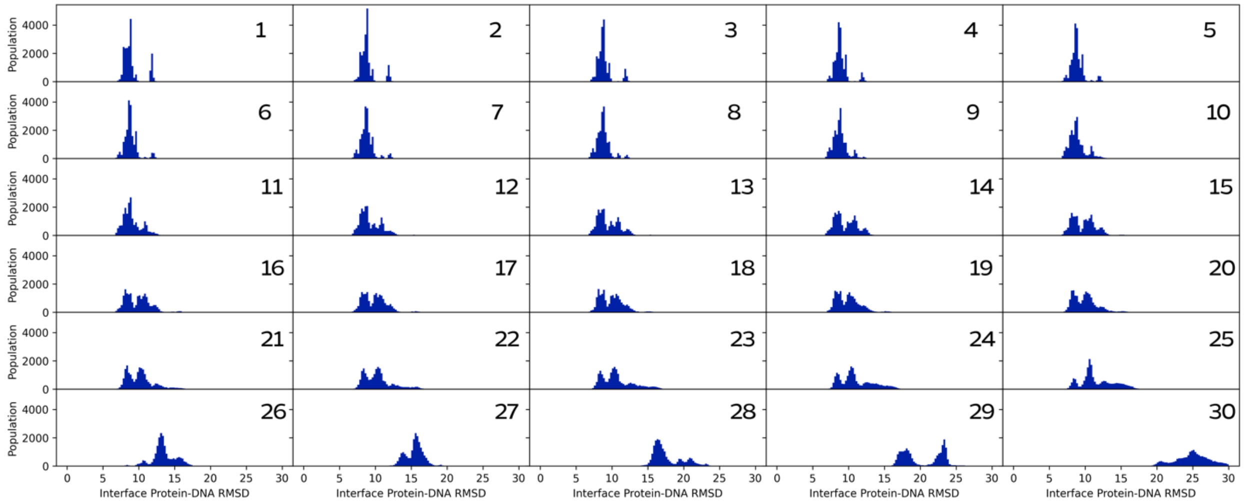

Figure S12. Histogram of ensemble RMSDs for 2B0D. Each panel corresponds to a different replica conditions (indicated by the number in the panel). Replica index 1 explores bound states at the lowest temperature while replica 30 explores unbound states at the highest temperature.

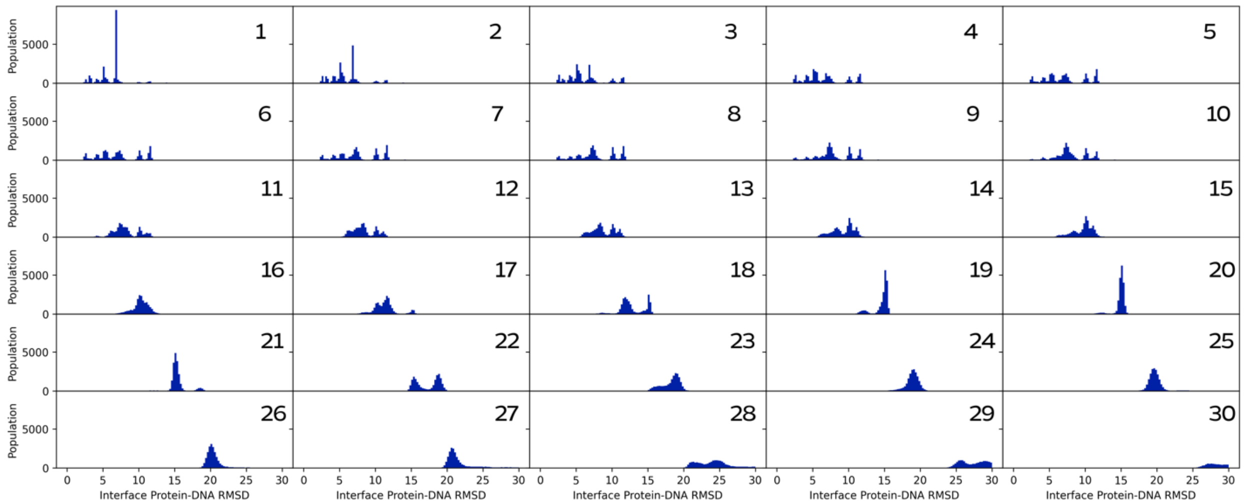

Figure S13. Histogram of ensemble RMSDs for 2DGC. Each panel corresponds to a different replica conditions (indicated by the number in the panel). Replica index 1 explores bound states at the lowest temperature while replica 30 explores unbound states at the highest temperature.

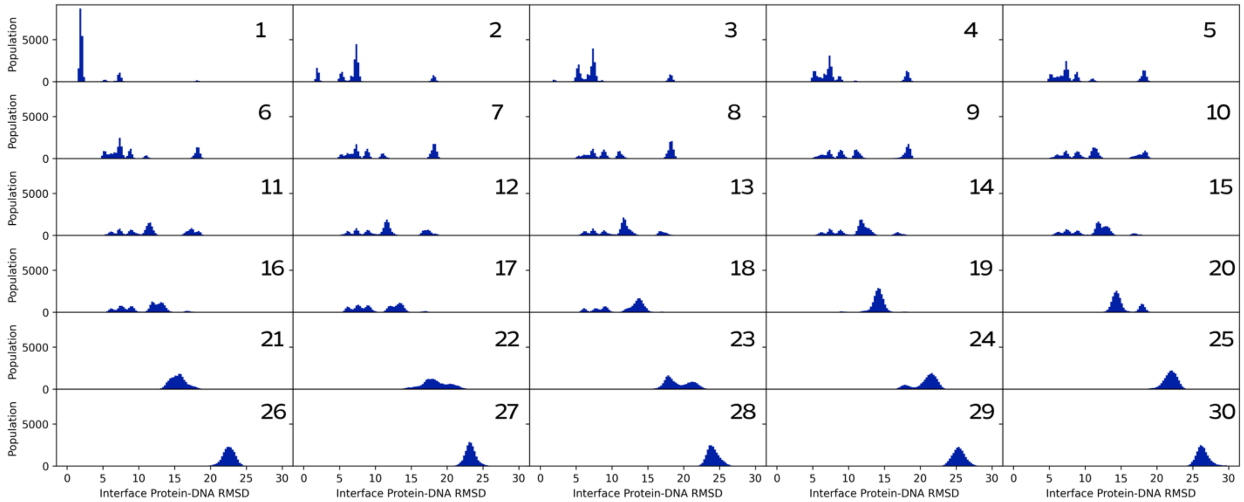

Figure S14. Histogram of ensemble RMSDs for 2R1J. Each panel corresponds to a different replica conditions (indicated by the number in the panel). Replica index 1 explores bound states at the lowest temperature while replica 30 explores unbound states at the highest temperature.

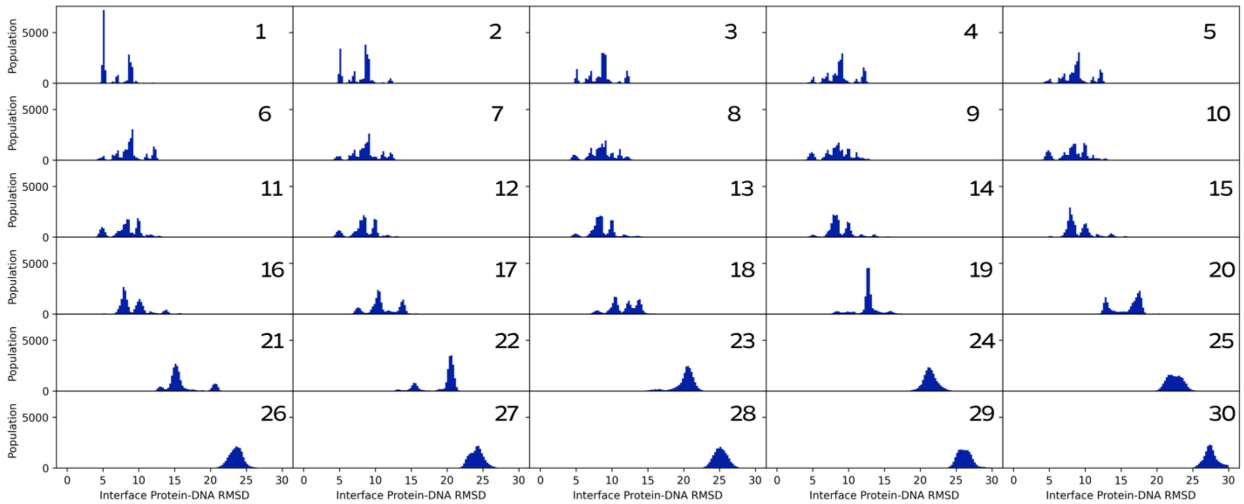

Figure S15. Histogram of ensemble RMSDs for 3CRO. Each panel corresponds to a different replica conditions (indicated by the number in the panel). Replica index 1 explores bound states at the lowest temperature while replica 30 explores unbound states at the highest temperature.

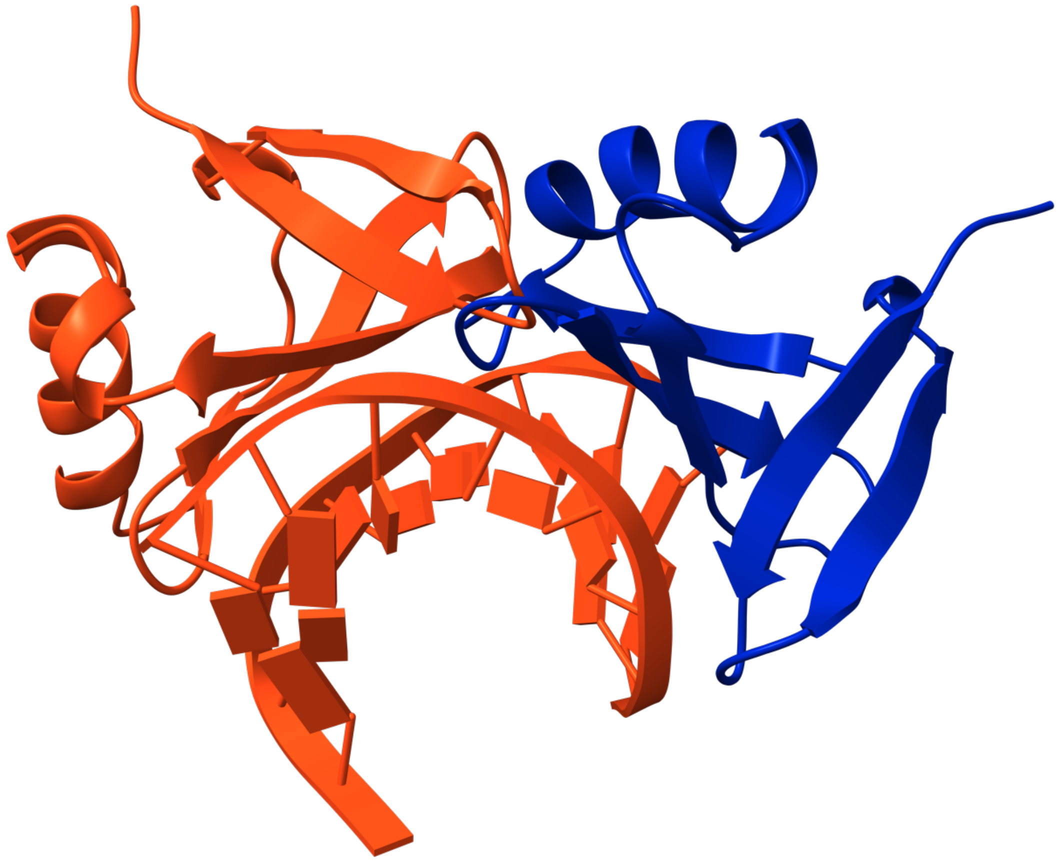

Figure S16. Alternative binding mode observed in 1AZP. Orange is reference, blue is the protein bound to the palindromic mirror of the binding site.

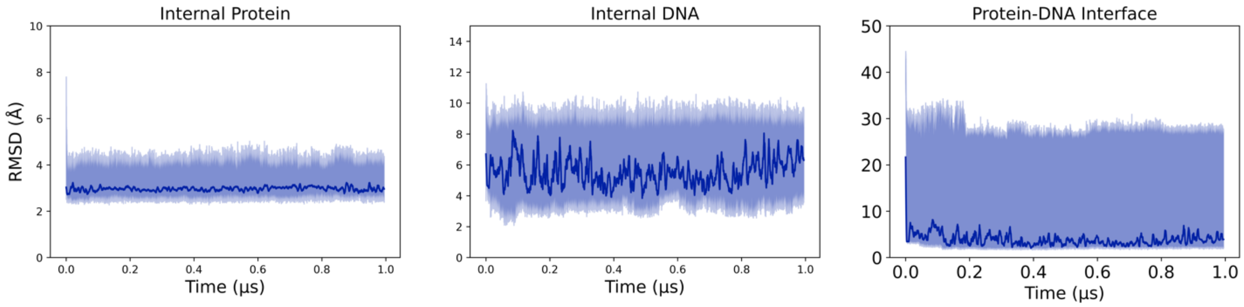

Figure S17. RMSD of protein, DNA, and protein-DNA interface for the lowest replica (blue line) averaged on a 100-frame window. The light background in each panel shows the min and max RMSD value of that frame across all replicas to emphasize the flexibility of the two binding partners. The data corresponds to the 1A74 system.

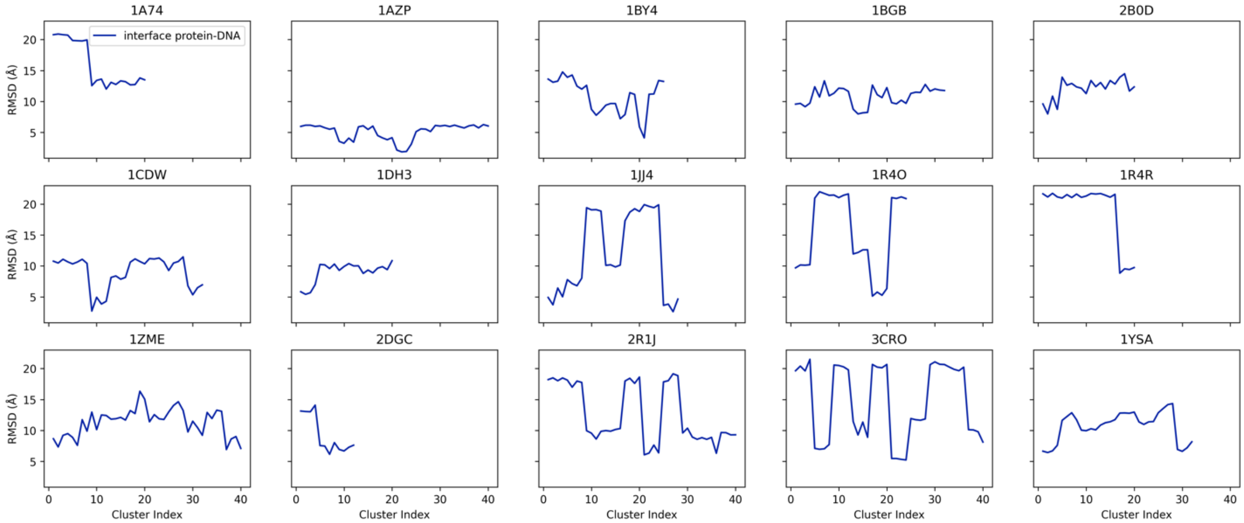

Figure S18. RMSD of clusters generated by HADDOCK. RMSD is taken from protein-DNA interface residues after aligning on the same mask (same protocol we used elsewhere in this work).

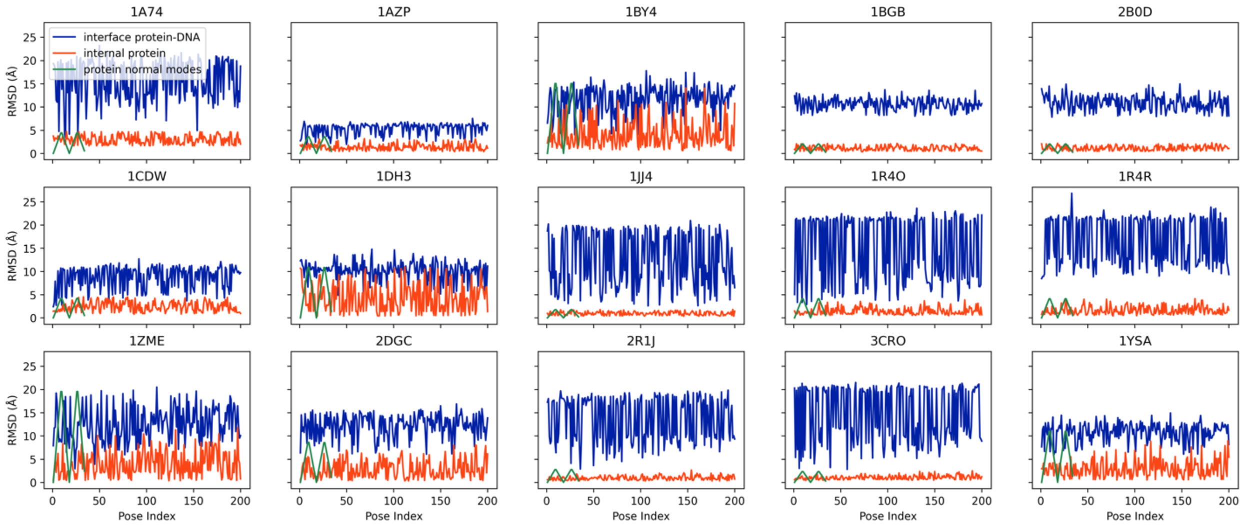

Figure S19. RMSD of all models generated by HADDOCK. Blue is the RMSD is taken from protein-DNA interface residues after aligning on the same mask (same protocol we used elsewhere in this work). Orange is internal protein RMSD, and green is the RMSD of initial protein structures provided to HADDOCK.

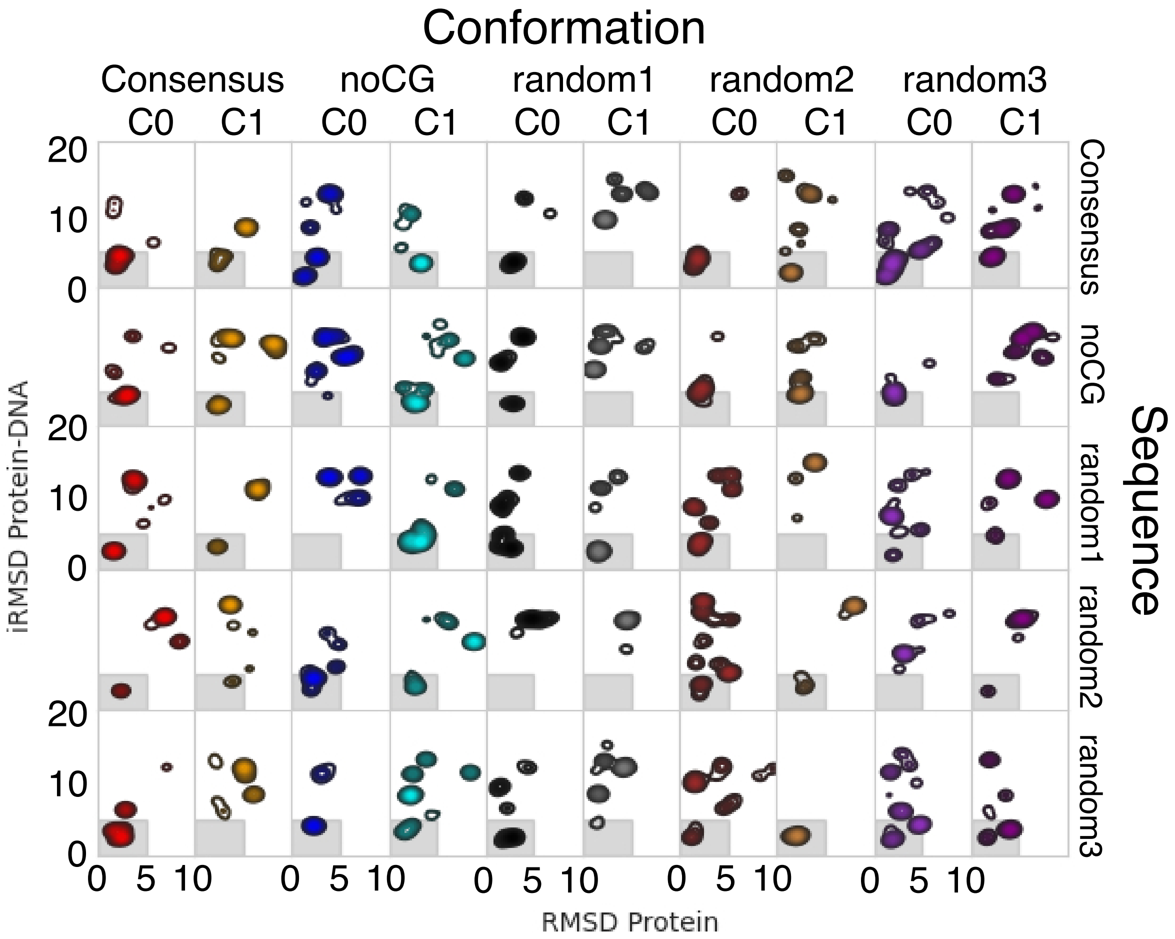

Figure S20. Multiple sequences and conformations are viable for Bzip-DNA binding. Each plot represents a particular sequence (denoted by the row, see labels on the right) and DNA conformation (denoted by column, belonging to clusters from free DNA simulations on each sequence). Each plot represents the interface RMSD vs the protein RMSD. MELD-DNA allows conformational freedom to the protein, which can sample a diverse set of conformations, but is mostly found in its native-like conformation when binding -- either at the correct binding site (grey box area) or others, denoted by protein RMSD below 5Å.

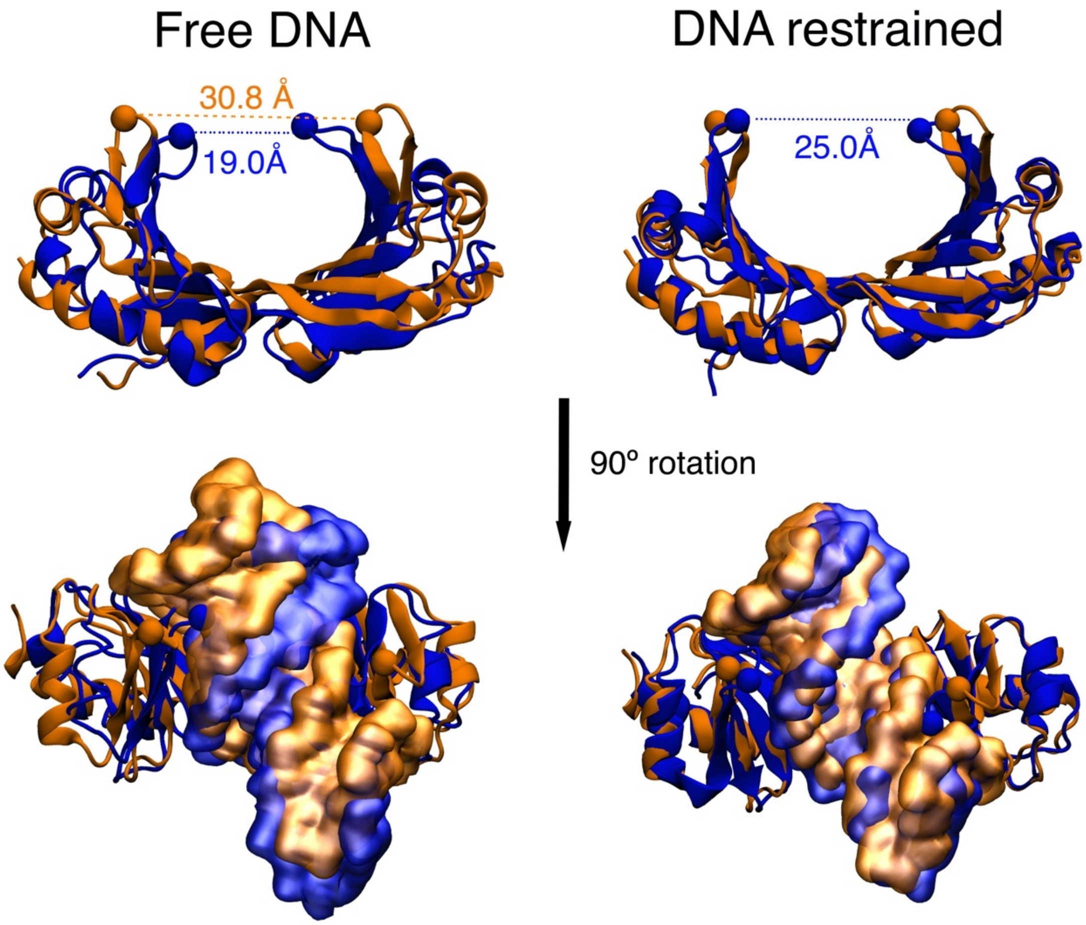

Figure S21. The TATA binding protein is highly flexible, modulating its ability to wrap around DNA's major groove. Native (orange) and simulated (blue) binding of the TATA box binding protein (cartoon representation) to DNA (space filling). In free simulations, the loop regions in the protein wrap more tightly around the DNA structure inducing a larger deformation (left). When the DNA is restrained with flat-bottom harmonic restraints, the protein still wraps around the major groove more tightly than in the experimental structure.

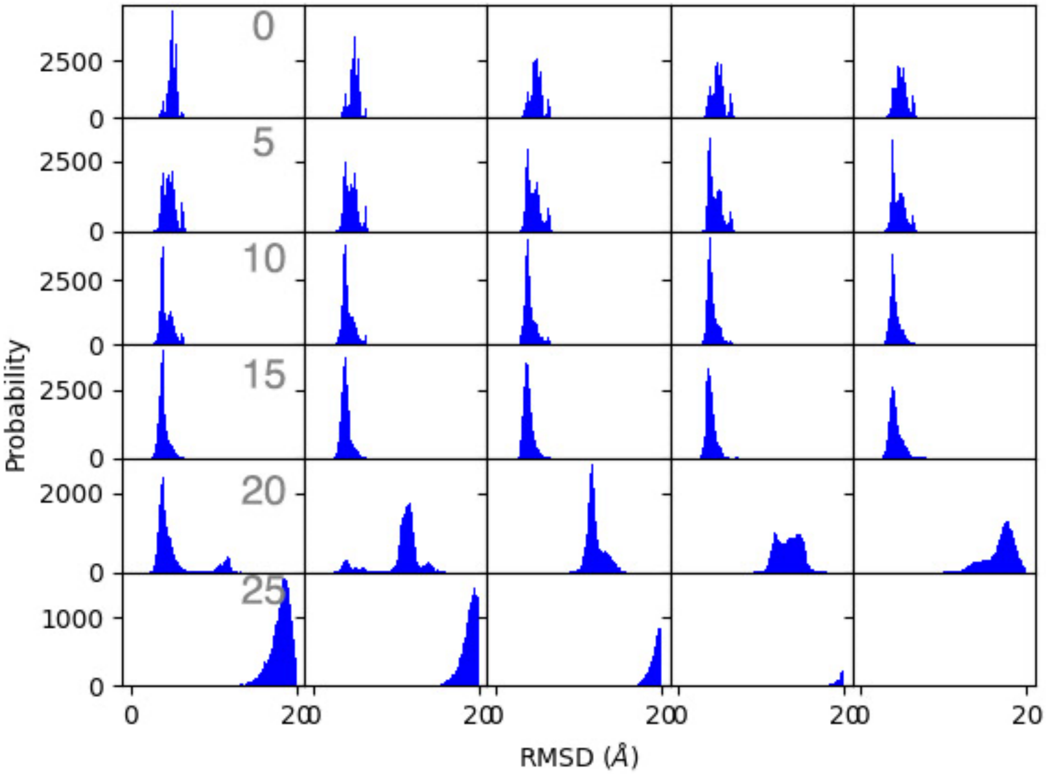

Figure S22. Protein-DNA RMSD distribution for each replica in the Replica Exchange ladder for the TATA system. Replica number increases to the right. Low replica index maps to low temperature and strong enforcement of the data (bound/misbound states). High replica indexes map to high temperature and vanishing restraints, enabling sampling of unbound states. Surprisingly, the most native-like conformations are identified in replicas 11-20, which show the lowest RMSD to native – this state has low population at the lowest replicas.

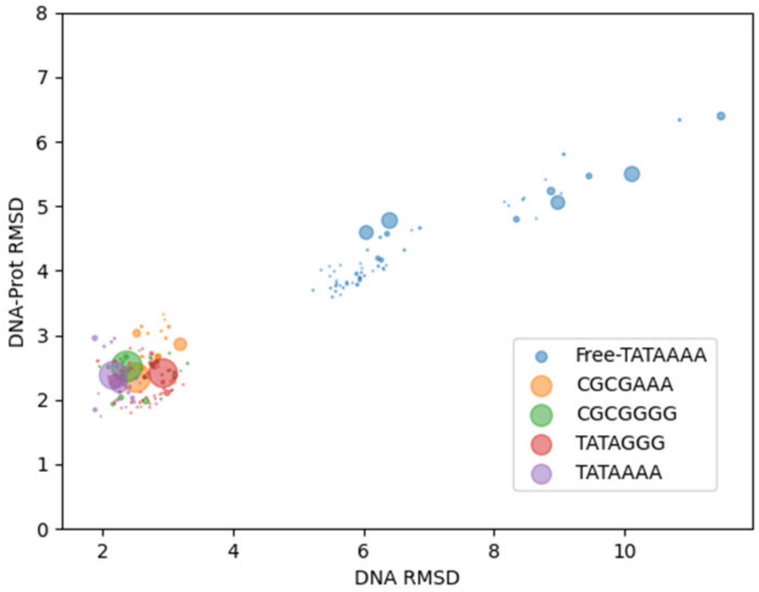

Figure S23. Accuracy of the top cluster in TATA-box binding simulations. Each simulation keeps the DNA restrained to the bound conformation except for the consensus sequence which is simulated restrained (purple) and starting from B-DNA (blue). Each cluster is represented as a dot, with population proportional to the size of the dot. When the DNA is able to deform, the protein wraps around the DNA major Groove more tightly, resulting in larger protein and DNA RMSD with respect to the restrained simulations.

SI References

1. Jorgensen,W.L., Chandrasekhar,J., Madura,J.D., Impey,R.W. and Klein,M.L. (1983) Comparison of simple potential functions for simulating liquid water. *J Chem Phys*, 79, 926–935.

2. Ivani,I., Dans,P.D., Noy,A., Pérez,A., Faustino,I., Hospital,A., Walther,J., Andrio,P., Goñi,R., Balaceanu,A., *et al.* (2016) Parmbsc1: a refined force field for DNA simulations. *Nat Methods*, 13, 55–58.

3. Joung,I.S. and Cheatham,T.E. (2008) Determination of Alkali and Halide Monovalent Ion Parameters for Use in Explicitly Solvated Biomolecular Simulations. *J Phys Chem B*, 112, 9020–9041.

4. Macke,T.J. and Case,D.A. (1997) Modeling Unusual Nucleic Acid Structures. *Acs Sym Ser*, 10.1021/bk-1998-0682.ch024.

5. MacCallum,J.L., Perez,A. and Dill,K. (2015) Determining protein structures by combining semireliable data with atomistic physical models by Bayesian inference. *Proc National Acad Sci*, 112, 6985–6990.

6. Fukunishi,H., Watanabe,O. and Takada,S. (2002) On the Hamiltonian replica exchange method for efficient sampling of biomolecular systems: Application to protein structure prediction. *J Chem Phys*, 116, 9058–9067.

7. Daura,X., Gademann,K., Jaun,B., Seebach,D., Gunsteren,W.F. van and Mark,A.E. (1999) Peptide Folding: When Simulation Meets Experiment. *Angewandte Chemie Int Ed*, 38, 236–240.

8. Perez,A., MacCallum,J.L. and Dill,K.A. (2015) Accelerating molecular simulations of proteins using Bayesian inference on weak information. *P Natl Acad Sci Usa*, 112, 11846–51.

9. III,T.E.C., Cieplak,P. and Kollman,P.A. (1999) A Modified Version of the Cornell et al.Force Field with Improved Sugar Pucker Phases and Helical Repeat. *J Biomol Struct Dyn*, 16, 845–862.

10. Perez,A., Marchan,I., Svozil,D., Sponer,J., Cheatham,T.E., Laughton,C.A. and Orozco,M. (2007) Refinement of the AMBER force field for nucleic acids: improving the description of alpha/gamma conformers. *Biophys J*, 92, 3817–3829.

11. Maier,J.A., Martinez,C., Kasavajhala,K., Wickstrom,L., Hauser,K.E. and Simmerling,C. (2015) ff14SB: Improving the Accuracy of Protein Side Chain and Backbone Parameters from ff99SB. *J Chem Theory Comput*, 11, 3696–3713.

12. Hornak,V., Abel,R., Okur,A., Strockbine,B., Roitberg,A. and Simmerling,C. (2006) Comparison of multiple Amber force fields and development of improved protein backbone parameters. *Proteins Struct Funct Bioinform*, 65, 712–725.

13. Nguyen,H., Roe,D.R. and Simmerling,C. (2013) Improved Generalized Born Solvent Model Parameters for Protein Simulations. *J Chem Theory Comput*, 9, 2020–2034.

14. Nguyen,H., Perez,A., Bermeo,S. and Simmerling,C. (2015) Refinement of Generalized Born Implicit Solvation Parameters for Nucleic Acids and Their Complexes with Proteins. *J Chem Theory Comput*, 11, 3714–3728.

15. Morrone,J.A., Perez,A., MacCallum,J. and Dill,K.A. (2017) Computed Binding of Peptides to Proteins with MELD-Accelerated Molecular Dynamics. *J Chem Theory Comput*, 13, 870–876.

16. Morrone,J.A., Perez,A., Deng,Q., Ha,S.N., Holloway,M.K., Sawyer,T.K., Sherborne,B.S., Brown,F.K. and Dill,K.A. (2017) Molecular Simulations Identify Binding Poses and Approximate Affinities of Stapled α-Helical Peptides to MDM2 and MDMX. *J Chem Theory Comput*, 13, 863–869.
